# Gene activation by dCas9-CBP and the SAM system differ in target preference

**DOI:** 10.1038/s41598-019-54179-x

**Published:** 2019-12-02

**Authors:** Suresh Sajwan, Mattias Mannervik

**Affiliations:** Department of Molecular Biosciences, The Wenner-Gren Institute, Stockholm University, 10691 Stockholm, Sweden

**Keywords:** Reverse transcription polymerase chain reaction, Transcription, Drosophila, Histone post-translational modifications, Reverse transcription polymerase chain reaction

## Abstract

Gene overexpression through the targeting of transcription activation domains to regulatory DNA via catalytically defective Cas9 (dCas9) represents a powerful approach to investigate gene function as well as the mechanisms of gene control. To date, the most efficient dCas9-based activator is the Synergistic Activation Mediator (SAM) system whereby transcription activation domains are directly fused to dCas9 as well as tethered through MS2 loops engineered into the gRNA. Here, we show that dCas9 fused to the catalytic domain of the histone acetyltransferase CBP is a more potent activator than the SAM system at some loci, but less efficient at other locations in *Drosophila* cells. Our results suggest that different rate-limiting steps in the transcription cycle are affected by dCas9-CBP and the SAM system, and that comparing these activators may be useful for mechanistic studies of transcription as well as for increasing the number of hits in genome-wide overexpression screens.

## Introduction

The type II clustered, regularly interspaced, short palindromic repeat/CRISPR-associated protein 9 (CRISPR-Cas9) system is a versatile genome engineering tool, and the development of nuclease-deactivated Cas9 (dCas9) fusion proteins allow for targeting of effector domains to virtually any genomic region^1^. Fusion of transcription activation or repression domains to dCas9 has been used to precisely modulate gene expression from gene promoters, both in cell culture and in *Drosophila*^2,3^. dCas9 can also be fused to histone or DNA-modifying protein domains. Such EpiEffectors have been used to identify and characterize functional regulatory elements in mammalian cells^1^. One such EpiEffector, dCas9-p300^core^ has been used to target proximal and distal regulatory regions in mammalian cell lines^4^, and has been utilized in gain of function screens for cis-regulatory element activity^5^. The p300 core domain has also been fused directly to CRISPR/Cpf1^6^, and adopted as a component of the CRISPR-Cas9 based Casilio and SAM systems where it is recruited through modified gRNAs^7,8^. Here, we describe a *Drosophila* dCas9-CBP EpiEffector that proved to be more efficient in activating some genes than the Synergistic Activation Mediator (SAM) system that targets three different transcription activation domains to the genome^9^. *Drosophila* CBP, also known as Nejire, is the sole homolog of the related mammalian CBP and p300 proteins^10^. CBP and p300 are acetyltransferases that target histone 3 lysine 27 (H3K27), H3K18, H4K8, several lysines in H2B, as well as many non-histone proteins^11,12^. CBP and p300 function as transcriptional co-regulators and interact with many different transcription factors^10^. Consequently, p300/CBP are present at many transcriptional enhancer sequences and p300/CBP ChIP-seq is a widely used approach to identify putative enhancers^13–15^. Interestingly, we find that both dCas9-CBP and SAM can function from a distance of tens of kb to activate gene expression, but genomic loci respond differently to these two activators. This indicates that dCas9-CBP and SAM target different rate-limiting steps in the transcription cycle, and suggests that dCas9-CBP could be useful for overexpressing genes that are refractory to activation by the SAM system.

## Results

### The CBP HAT domain fused to dCas9 outperforms a fusion to MS2 coat protein

**Figure 1:**
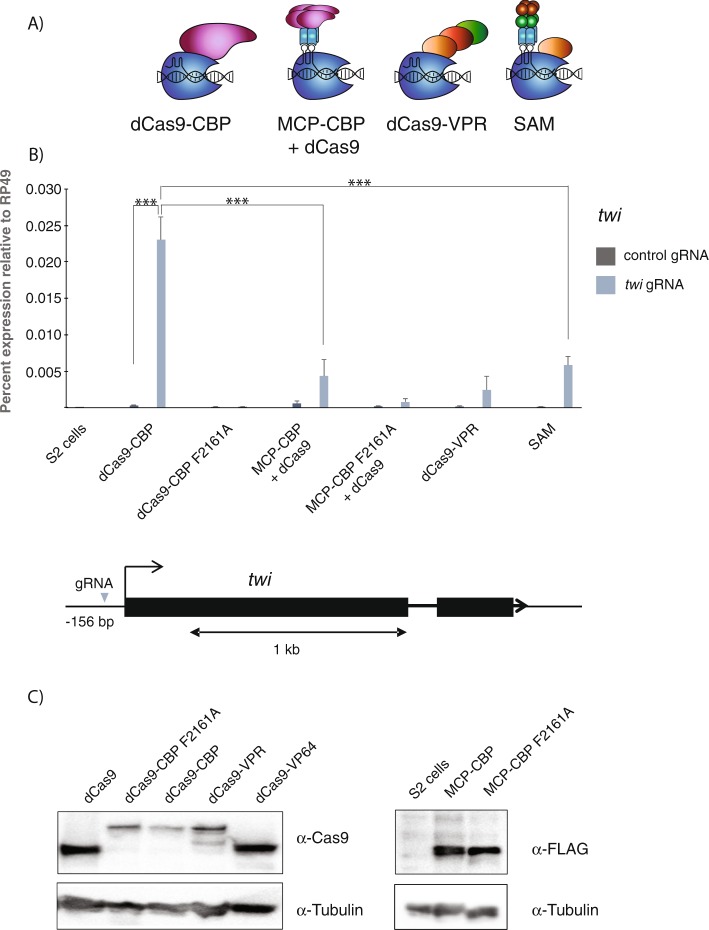
Direct fusion of the CBP HAT domain to dCas9 outperforms a MS2 coat protein-CBP fusion plus dCas9 combination. (**A**) Schematic drawings of dCas9 fused to the HAT domain of CBP, MS2 coat protein fused to the CBP HAT domain (MCP-CBP) where two MCP dimers recognize two MS2 loops in the gRNA and dCas9 thereby brings four CBP domains to the locus, dCas9-VPR where three activation domains are fused to dCas9, and the synergistic activation mediator (SAM) system where MCP targets eight activation domains to dCas9 fused with the VP64 activation domain. (**B**) RT-qPCR showing *twist* (*twi*) expression in *Drosophila* S2 cells and in S2 cells transfected with UAS-dCas9 fusions or UAS-dCas9 and UAS-MCP fusions together with *Actin*-Gal4 in the presence of a control gRNA or *twi* promoter gRNA. Expression is plotted relative to *RP49*. n = 3 biological replicates and error bars represent S.E.M. One-way ANOVA with post hoc Tukey test was used to calculate statistically significant differences. The full statistical analysis and fold activation in relation to the control (QUAS) gRNA is shown in Supplementary Table 2. The F2161A mutation disrupts the catalytic function of the CBP HAT domain. Below the graph is a schematic drawing of the *twi* locus. (**C**) Western blot showing expression of the dCas9 and MCP fusion proteins. Uncropped images are shown in Supplemental Fig. S9.

In order to develop an efficient system for engineering the chromatin state of *Drosophila* cells, we compared a direct fusion of CBP’s histone acetyltransferase (HAT)-domain to catalytically dead Cas9 (dCas9), with fusions to the MS2 coat protein (MCP). In both cases, the fusions included the bromo-, RING-, PHD-, and catalytic domains from CBP (amino acids 1696–2329, corresponding to the p300 core domain used in mammalian cells^4^). In the synergistic activation mediator (SAM) system^9^, MCP fused to the p65 and HSF-1 activation domains is combined with dCas9-VP64 and modified gRNAs containing two MS2 loops (Fig. 1A). This system has proven to be the most efficient for gene activation through dCas9 ^2,3^. We therefore fused the catalytic domain of CBP to MCP or directly to dCas9, and compared them to the SAM system and to dCas9-VPR where three activation domains are fused directly to dCas9 ^16^. We also introduced a point mutation (F2161A) in the CBP catalytic core that we previously showed disrupts the catalytic activity of CBP^17^. These different constructs were placed under UAS promoters and transiently transfected into S2 cells together with actin5C-Gal4 activator and a gRNA with MS2 loops that targets the *twist* (*twi*) gene promoter (Fig. 1B). We harvested RNA at different time points after transfection and found robust gene activation at 48 h post transfection (Supplementary Fig. S1). As shown previously^2^, both the SAM system and dCas9-VPR could activate endogenous *twi* expression (Fig. 1B). Surprisingly, dCas9-CBP was even better at activating *twi* (Fig. 1B), despite the nine activation domains targeted to the promoter in the SAM system and only one CBP domain fused to dCas9. We used one-way ANOVA with post hoc Tukey test to calculate statistically significant differences between the transfections (for the full statistical analysis, see Supplementary Table S2), showing that dCas9-CBP is a significantly better activator than SAM at the *twi* promoter. The dCas9-CBP F2161A protein failed to activate *twi*, indicating that CBP HAT activity is necessary for this gene activation.

When we combined MCP-CBP with dCas9, there was a non-specific effect on transcription, as *twi* RNA levels slightly increased with a negative control *QUAS* gRNA. In the presence of *twi* gRNA, expression increased further, but not to the same level as with dCas9-CBP (Fig. 1B). At another locus, *engrailed* (*en*), dCas9-CBP is also significantly more potent than MCP-CBP combined with dCas9, and the MCP-CBP fusion caused increased expression in the absence of specific gRNA at several loci (only statistically significant at the *en* locus, Supplementary Fig. S2 and Table S2). These results are consistent with a study demonstrating that dCas9-p300 activates the *IL1RN* promoter more efficiently than MCP-p300 in mammalian cells^8^. Since the MS2 loops in the gRNA are not needed for dCas9-CBP function, we used otherwise identical gRNAs with and without the MS2 loops. This showed that dCas9-CBP activated *en* slightly better together with gRNAs that lack the MS2 loops (Supplementary Fig. S3). A Western blot showed that differences in protein levels cannot explain why dCas9-CBP is the most efficient activator of *twi* expression (Fig. 1C). We conclude that dCas9-CBP is a potent transcription activator that outperforms a system of dCas9 combined with MCP-CBP.

### dCas9-CBP and the SAM system differ in what genes can be strongly activated

**Figure 2:**
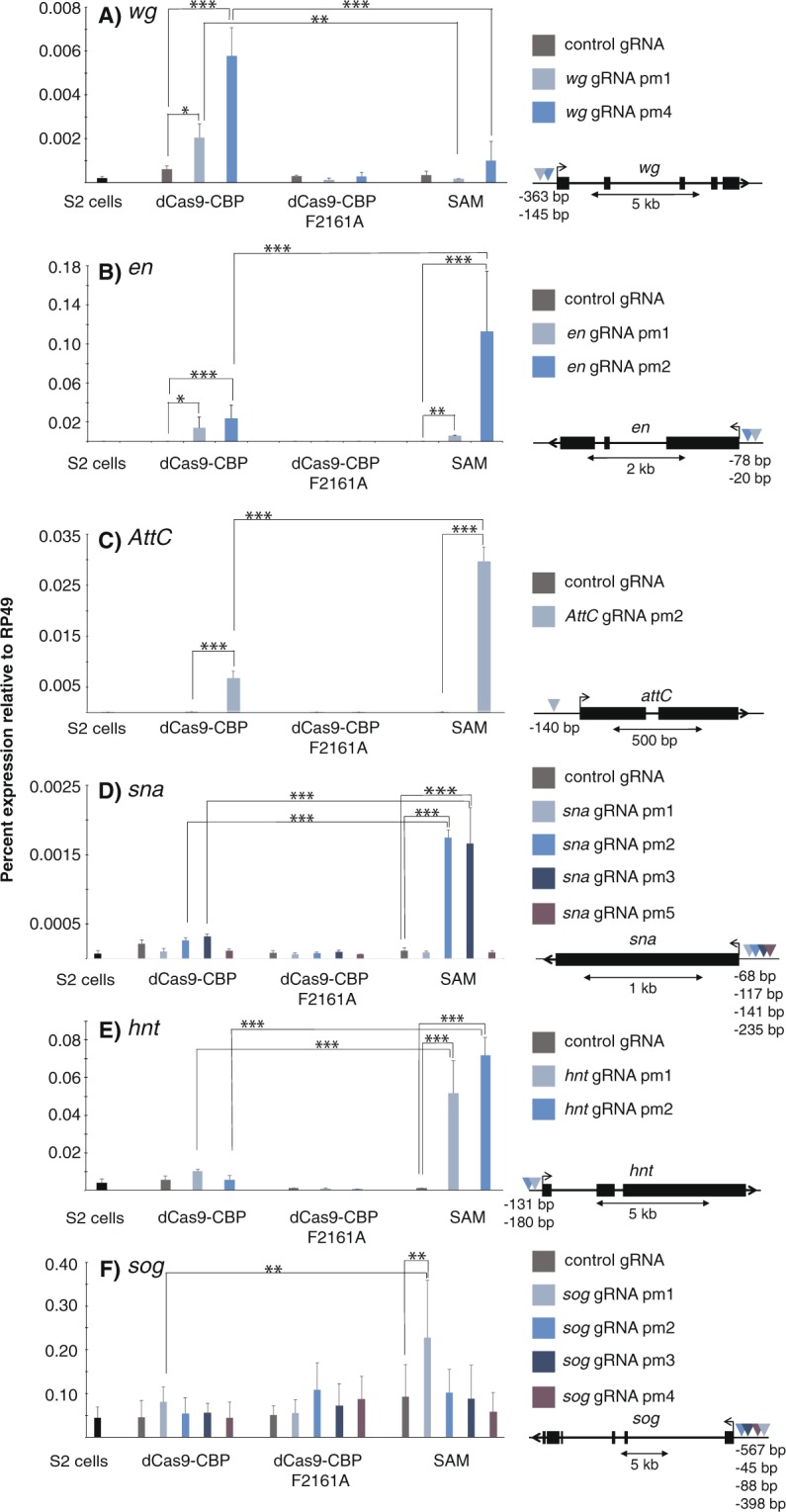
The SAM system and dCas9-CBP differ in target preference. Expression of (**A**) *wingless (wg)* (**B**) *engrailed (en)* (**C**) *Attacin C (AttC)* (**D**) *snail (sna)* (**E**) *hindsight (hnt)*, and (**F**) *short gastrulation (sog)* relative to *RP49* in S2 cells transfected with dCas9 fusions and control gRNA or gRNAs targeting the promoters of these genes as measured by RT-qPCR. Statistically significant differences are calculated as in Fig. 1, n = 3 (*wg, en, AttC*) or 2 (*sna, hnt, sog*), error bars represent S.E.M. Schematic drawings of the loci and location of the gRNAs relative to the transcription start sites (TSS) are shown to the right.

We next compared dCas9-CBP with the SAM system at other gene loci in S2 cells. We found that dCas9-CBP could weakly activate the *wingless* (*wg*) promoter, whereas the SAM system did not have a statistically significant effect on this gene (Fig. 2A). By contrast, although *en* and *Attacin-C (AttC)* expression were also activated by dCas9-CBP when targeted to the respective promoter, this was not as efficient as the SAM system (Fig. 2B,C). This indicates that a different step in the transcription cycle is rate-limiting at these genes compared to *twi* and *wg*, where dCas9-CBP is a more potent activator than the SAM system. When targeted to the promoters of the *snail* (*sna*), *hindsight* (*hnt*), and *short-gastrulation* (*sog*) genes, dCas9-CBP failed to activate transcription whereas the SAM system could activate them weakly (Fig. 2D–F). The *hnt* and *sog* genes are already moderately expressed in S2 cells, and are not further activated by dCas9-CBP. Taken together, these results indicate that dCas9-CBP is able to activate non- or lowly-expressed genes in S2 cells but not genes that are already expressed.

### Both dCas9-CBP and SAM can activate transcription from a distance

**Figure 3:**
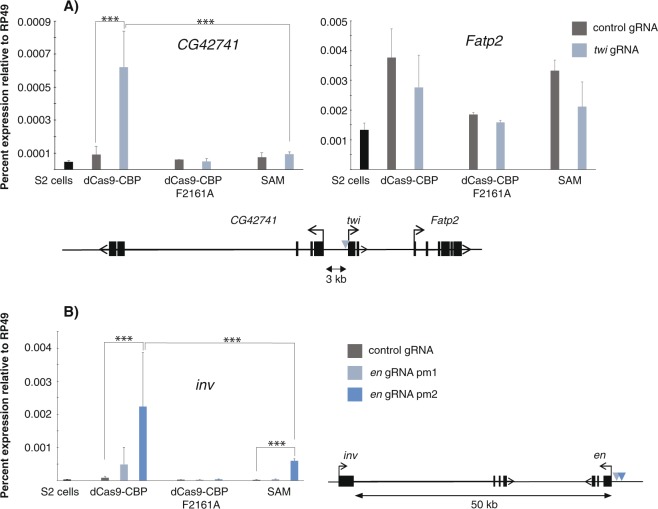
Promoter-bound dCas9-CBP and SAM can activate nearby genes. (**A**) *CG42741* and *Fatp2* expression in S2 cells transfected with dCas9 fusions and control or gRNA targeting the *twi* promoter (same as in Fig. 1B). (**B**) Expression of *invected (inv)* in S2 cells transfected with dCas9 fusions and control or gRNAs targeting the *en* promoter (same as in Fig. 2B) located more than 50 kb from the *inv* TSS. Statistical significance is indicated. n = 2 (*CG42741*) or 3 (*inv*) and error bars display S.E.M. Schematic drawings of the loci are shown.

Since CBP is well known for its role at enhancers^10^, we investigated whether dCas9-CBP could act at a distance. First we turned to genes that flank the silent loci that are activated by promoter-bound dCas9-CBP. This showed that expression of *CG42741* that is located 3.2 kb from the *twi* transcription start site (TSS) could be weakly activated by dCas9-CBP bound to the *twi* promoter (Fig. 3A). *CG42741* is poorly expressed in S2 cells and is up-regulated by dCas9-CBP but not by the SAM system, whereas *Fatp2* that is moderately expressed in untreated cells is not activated by dCas9-CBP. Strikingly, the *invected* (*inv*) gene whose promoter is located more than 50 kb from the *en* promoter could be activated by dCas9-CBP with *en* gRNA (Fig. 3B). The SAM system targeted to the *en* promoter also activated *inv*, but to a lower level than dCas9-CBP, despite being a stronger activator of *en* than dCas9-CBP (Fig. 3B, compare to Fig. 2B). It is possible that activation of flanking genes is the result of off-target activity of the dCas9 fusions, but we favor the idea of activation at a distance since the relative activity of dCas9-CBP and SAM differ at *en* and *inv*. At this locus, dCas9-CBP acts more efficiently over a large distance than the SAM system. However, expression of the genes flanking the *wg* and *AttC* loci were not affected by either dCas9-CBP nor by SAM targeted to the respective promoter (Supplementary Fig. S4).

**Figure 4:**
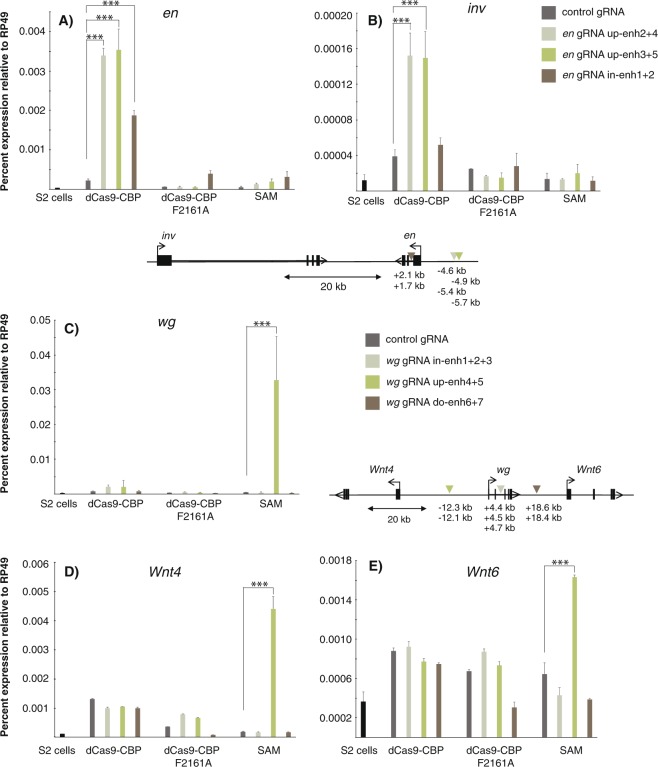
Enhancer-bound dCas9-CBP and SAM can activate transcription from a distance. RT-qPCR showing *en* (**A**) *inv* (**B**) *wg* (**C**) *Wnt4* (**D**) and *Wnt6* (**E**) expression in S2 cells transfected with dCas9 fusions and gRNAs that either target upstream or intronic *en* enhancers or that target intronic, upstream or downstream *wg* enhancers. Schematic drawings of the loci and locations of the gRNAs are shown. n = 2. Error bars show S.E.M., and statistical significance calculated as in Fig. 1.

Next, we targeted dCas9-CBP to enhancer regions. Multiple enhancers control *en* and *inv* expression in embryos and in imaginal discs^18^. We designed gRNAs from defined intronic and upstream enhancer regions that both have been shown to generate a 15-stripe pattern in embryos^18,19^. We found that dCas9-CBP could activate *en* expression from both locations, but more strongly from the upstream region located ~5 kb from the TSS (Fig. 4A). dCas9-CBP could also weakly activate *inv* expression from this upstream enhancer (Fig. 4B). By contrast, the SAM system targeted to these enhancers had no effect on *en* and *inv* expression (Fig. 4A,B).

This is different from a *wg* enhancer that can be activated by SAM but not by dCas9-CBP (Fig. 4C). We targeted three putative *wg* enhancers with multiple gRNAs, one intronic enhancer 4.5 kb from the TSS, one located 18 kb downstream of the TSS, and one 12 kb upstream from the TSS. These enhancers were identified in S2 cells by the STARR-seq assay^20^. Whereas dCas9-CBP had no effect on *wg* expression from either of these enhancers, the SAM system efficiently activated *wg* from the upstream enhancer (Fig. 4C). The SAM system also activated the *Wnt4* gene and had a weak effect on *Wnt6* from this position, whereas dCas9-CBP did not influence expression of these genes (Fig. 4D,E). Interestingly, although SAM could activate *wg* from an enhancer, it was a very poor activator at the promoter (compare Fig. 4D with Fig. 2A). By contrast, the *en* promoter is more potently activated by SAM than by dCas9-CBP, but SAM fails to activate *en* from an enhancer that is activated by dCas9-CBP (compare Fig. 4A with Fig. 2B). Thus, genes do not only respond differently to different types of activators, but the genomic position also influences which fusion-protein that is able to activate transcription.

We also used gRNAs to defined *twi* embryonic enhancers^19^ as well as to more enhancers that are active in S2 cells according to the STARR-seq assay^20^ (see Supplementary Fig. S5 for their location and chromatin state). In none of these cases did we observe activation of the corresponding gene (Supplementary Fig. S6). We conclude that both dCas9-CBP and the SAM system can activate transcription from a distance of at least several tens of kb. However, they do not work from any position in the genome.

### Chromatin state and core promoter motifs may influences dCas9-mediated gene activation

In order to investigate the difference between genomic regions that activate transcription when bound by dCas9-fusion proteins and those that do not, we compared the chromatin state between these sites in untreated cells by available modENCODE ChIP-chip data^21^. We used H3K27 acetylation (H3K27ac) as a mark of active enhancers and promoters, and H3K27 tri-methylation (H3K27me3) as a mark of Polycomb-silenced regions. Both poorly and moderately expressed genes (*twi*, *wg*, *en*, *AttC, sna, hnt, sog* and *Snoo*) are decorated with H3K27me3 to different extents in S2 cells, but only the promoters of the moderately expressed genes *hnt, sog* and *Snoo* are enriched for H3K27ac (Supplementary Fig. S5). So are the *wg* upstream and downstream enhancers and the STARR-seq enhancers of *sog* and *Snoo* (Supplementary Fig. S5). All regions from which dCas9-CBP activates transcription lack H3K27ac, whereas SAM can activate also from certain regions with some H3K27ac, indicating that only hypoacetylated genomic regions may be responsive to dCas9-CBP. However, dCas9-CBP does not activate from all hypoacetylated regions, e.g. from the *sna* promoter and *wg* intronic enhancer. We conclude that chromatin state may influence dCas9-CBP and SAM-mediated activation, but that it cannot by itself predict from which genomic positions these fusion-proteins will regulate transcription.

To further examine the link between acetylation and gene activation at the targeted regions, we explored if acetylation differences between cell types correlate with dCas9-CBP activity, and compared H3K27ac in S2 cells with early (0–4 h old) embryos (Table S3). No consistent difference could be seen for regions that respond to dCas9-CBP and regions that do not.

We then considered that core promoter elements may influence the response to dCas9 fusion proteins. Using information from the ElemeNT database^22^, we listed the motifs that are present in gene promoters. Interestingly, the *sna, hnt*, and *sog* genes all contain a TATA-box and do not respond to dCas9-CBP, whereas *twi, wg* and *en* that are activated by dCas9-CBP lack a TATA-box but contain the Initiator (Inr) motif (Table 1). The *AttC* promoter is an exception, since it contains a TATA-box but can be activated by dCas9-CBP. The core promoter may thus affect gene activation by dCas9 fusions, but does not fully explain the difference between genes that are responsive and those that are not. We suggest that gene expression level, core promoter elements and chromatin state all influence the response to dCas9-CBP.

**Table 1:** List of gene expression, histone acetylation and core promoter elements.

Gene	% expression in untreated S2 cells relative to RP49 (n = 2–3, mean ± SEM)	H3K27ac in S2 cells				Core promoter elements	Activated by dCas9-CBP
		promoter	enhancer	enhancer	enhancer		
*twi*	0.00005 (0.00001)	−	+			Inr, DPE	+
*wg*	0.00020 (0.00007)	−	++	++	−	Inr	+
*en*	0.00005 (0.00001)	−	−	−		Inr, DPE	+
*AttC*	0.00009 (0.00001)	−				TATA, Inr, DPE	+
*sna*	0.00008 (0.00006)	−				TATA	−
*hnt*	0.00411 (0.00204)	+				TATA, Inr	−
*sog*	0.07711 (0.02106)	+	++			TATA, Inr, DPE	−
*Snoo*	0.09008 (0.00519)	++	++			TATA	−
**Nearby gene**							
*CG42741 (twi)*	0.00004 (0.000009)	−				Inr	+
*Fatp2 (twi)*	0.00133 (0.00022)	−				Inr, DPE	−
*inv (en)*	0.00002 (0.000008)	−				Inr, DPE	+
*Wnt4 (wg)*	0.00064 (0.00053)	−				Inr, DPE	−
*Wnt6 (wg)*	0.00102 (0.00066)	+				Inr	−
*CG43691 (AttC)*	0.00026 (0.00007)	+				TATA, Inr	−
*CG4744 (AttC)*	0.00025 (0.00007)	−				Inr	−

Given that CBP catalytic activity is required for gene activation in all cases examined (Figs. 1–4), we used ChIP-qPCR to investigate if targeting dCas9-CBP to the genome results in the expected histone hyperacetylation. However, we failed to detect a change in H3K27ac at both the *twi* promoter and *wg* upstream enhancer. Since this may be due to a low efficiency of the transient transfections, we generated stable cell lines expressing all components. Still no increase in H3K27ac could be detected (Supplementary Fig. S7). It is possible that only a small subset of cells in the population respond to dCas9-CBP, and that increased acetylation is masked by non-responsive cells. Alternatively, CBP may acetylate a different substrate that is needed for transcription activation.

### Gene activation *in vivo*

**Figure 5:**
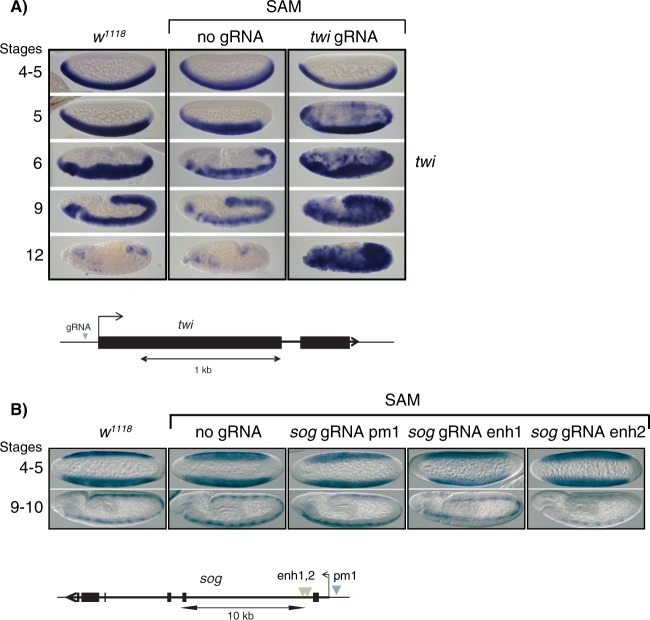
Gene activation in the early *Drosophila* embryo. (**A**,**B**) Maternally loaded SAM can activate zygotic *twi* expression but fails to activate *sog*. (**A**) Females containing UASp-dCas9-VP64, UASp-MCP-p65-HCF1 (SAM) and *alpha-tub*-Gal4 were crossed to males without gRNA (*w*^1118^) or to males expressing *twi* promoter gRNA, and 2–5 h old embryos were collected and hybridized with digoxigenin-labeled *twi* anti-sense probe. Wild-type (*w*^1118^) control embryos are shown to the left. Expression of *twi* is restricted to the mesoderm in *w*^1118^ (left) and in SAM containing embryos without gRNA (middle), but ectopically expressed in SAM embryos with *twi* gRNA (right). Only some cells ectopically express *twi* at stage 5, but many more cells activate *twi* at stages 6–12. (**B**) Same as (**A**) except that SAM females were crossed to wild-type males or to males expressing *sog* promoter or *sog* enhancer gRNAs and hybridized with a *sog* anti-sense probe. No ectopic expression could be detected. Schematic drawings of the loci and location of the gRNAs are shown.

To see if we could obtain gene activation *in vivo*, we created transgenic flies. Since most genes that we have targeted in S2 cells are expressed in the early embryo, we developed a system that uses a maternal supply of dCas9. We cloned dCas9-fusions and MCP-fusion in the UASp-K10 promoter that can be activated in the female germline, integrated them on chromosomes 2 and 3, and crossed them with the *alpha-tubulin*-Gal4 driver that is active in the female germline. This leads to the deposition of dCas9-fusions and MCP-fusions in the oocyte, and we crossed these females with transgenic males that express gRNAs from the U6 promoter. Embryos collected from this cross were subjected to whole-mount *in situ* hybridization. As shown in Fig. 5, a maternal supply of dCas9-VP64 and MCP-p65-HSF1 (the SAM system) crossed with *twi* promoter gRNA-expressing males resulted in ectopic *twi* expression, whereas a cross to wild-type males did not. Compared to endogenous *twi* expressed in the mesoderm, there was a delay in the onset of ectopic *twi* expression in SAM containing embryos. In cellularized stage 5 embryos just prior to gastrulation, *twi* expression appeared in the dorsal ectoderm and at the poles in most of the embryos. In older embryos more cells acquired *twi* expression, and by stage 9 most of the cells expressed *twi* (Fig. 5A). We have previously demonstrated that *twi* is decorated with H3K27me3 in neuroectoderm and dorsal ectoderm^23^, indicating that repressive chromatin states present in non-mesodermal cells compete with the SAM system. Prolonged expression of the SAM system overcomes this repression and leads to *twi* transcription in most cells. By contrast, a maternal supply of the SAM system combined with *sog* promoter or *sog* enhancer gRNAs was not able to activate *sog* expression in the embryo (Fig. 5B). In this case, there are features of *sog* repression that cannot be overcome even by prolonged SAM system targeting.

We also made flies expressing MCP-CBP or dCas9-CBP. However, both MCP-CBP and dCas9-CBP caused sterility when combined with *alpha-tubulin*-Gal4. Thus, it appears that a high level of the CBP HAT domain is detrimental to oogenesis. We therefore used a number of other Gal4 drivers and crossed them with either UAST dCas9-CBP or UASp-K10 dCas9-CBP for somatic expression. We obtained phenotypes in the absence of gRNA in these crosses, suggesting that high levels of CBP cause non-specific gene expression alterations. Further work is required to find the appropriate *in vivo* conditions for CBP expression without affecting global gene expression.

The SAM system has previously been shown to efficiently activate genes in several *Drosophila* tissues^3^. Our results complement these findings and show that it can also be loaded maternally to activate genes in the early embryo, whereas strong dCas9-CBP expression is detrimental to oogenesis.

## Discussion

We have fused the CBP HAT domain to dCas9 or to MCP in order to establish a system for epigenetic engineering in *Drosophila*. Consistent with a report comparing dCas9-p300 to MCP-p300 in mammalian cells^8^, we found that dCas9-CBP outperforms MCP-CBP also in *Drosophila*. This suggests that a direct dCas9 fusion is the preferred choice in any species or cell type. Targeting CBP to a promoter or an enhancer is sufficient for gene activation at several genomic locations, and this transcriptional activation by dCas9-CBP depends on CBP’s catalytic activity. Although we were unable to detect increased histone acetylation at targeted loci, it remains possible that histone acetylation by dCas9-CBP results in more accessible chromatin that facilitates transcription. A recent report demonstrated that only a few percent of the cells in a population where all cells expressed a dCas9 fusion responded to the activator^24^. Thus, dCas9-CBP may activate transcription and induce hyperacetylation in only a small proportion of cells also in our case. Alternatively, CBP acetylates other proteins that stimulate transcription.

Our results establish that CBP HAT domain over-expression causes non-specific effects on gene expression. In S2 cells, transfection of the MCP-CBP fusion protein caused up-regulation of several genes in the absence of specific gRNAs. This was much less of a problem with transfected dCas9-CBP. However, transgenic expression of dCas9-CBP resulted in sterility and non-specific wing phenotypes with tub-Gal4 or vg-Gal4, respectively. Similarly, expression of dCas9-CBP in the eye was recently shown to cause a phenotype in the absence of gRNA^25^. Thus, finding appropriate conditions for CBP HAT domain expression *in vivo* awaits further experiments. One possibility is that CBP expression results in a global increase in acetylation of both histone and non-histone proteins, which in turn causes toxicity and non-specific effects on gene expression. Consistent with this idea, we found an increase of protein acetylation in cells transfected with catalytically active dCas9-CBP, and an even more dramatic rise in acetylation in MCP-CBP transfected cells (Supplementary Fig. S8). We conclude that over-expression of this HAT domain causes global changes to the acetylome, and that this needs to be considered when applying this EpiEffector. Interestingly, dCas9 fused to the catalytic domain of the DNA methyltransferase Dnmt3 was shown to cause a global increase in DNA methylation in mouse ES cells and in human cell lines, which was unaffected by the presence of gRNA^26^. It appears that both the CBP and Dnmt3 catalytic domains can cause widespread off-target activity, and that controlling the levels of these EpiEffectors may be key to their successful use in epigenetic engineering.

Despite this caveat, we found that dCas9-CBP is a strong and relatively specific activator of gene expression in S2 cells with a target preference distinct from the established SAM gene activation system. This difference in efficiency between targeting a HAT and targeting transcription activation domains to the same genomic locations suggest that different rate-limiting steps are being affected by these two approaches. This is supported by a study showing that activation by dCas9-p300 and the SAM system in HEK-293 cells differ in their sensitivity to small molecule inhibitors^8^. Whereas dCas9-p300 was inhibited by the BET bromodomain inhibitor JQ1, the SAM system was not. We have previously shown that endogenous CBP stimulates both recruitment of RNA polymerase II to promoters as well as release into productive elongation by facilitating transcription through the +1 nucleosome^27^. One possibility is that the rate-limiting step affected by the SAM system, but not by dCas9-CBP, is release from promoter-proximal pausing, since we found that this step is not affected by CBP inhibition^27^. Instead, chromatin decompaction through acetylation of histones or other proteins may be the step controlled by dCas9-CBP. Consistent with this notion, none of the promoters or enhancers in our study that are pre-marked with H3K27ac responded to dCas9-CBP. This suggests that histone acetylation state should be considered when directing dCas9-CBP to genomic regions for gene activation.

Interestingly, the composition of core promoter motifs may also influence the response, and it was recently shown that transcriptional coregulators display specificity for distinct types of core promoters^28^. Since core promoter composition can affect initiation and pausing^29^, the various steps in the transcription cycle that are targeted by dCas9-CBP or the SAM system are likely to be influenced by a combination of chromatin state and promoter sequence.

Our results suggest that prior knowledge of core promoter composition, histone acetylation state and degree of transcription may be useful for designing gRNAs that are likely to result in dCas9-CBP mediated gene activation. With an appropriate expression level that minimizes off-target acetylation, the dCas9-CBP activator may be a useful complement to genome-wide over-expression screens through the SAM system given the different target preferences of the two activators.

## Materials and Methods

### Molecular cloning

A plasmid for gRNAs with MS2 loops was constructed from plasmid sgRNA2.0 (Addgene 61427)^9^. An amplicon consisting of gRNA sequence complementary to the DNA target and core gRNA region with two MS2 loop sequences was cloned into the pCFD3 plasmid^30^, at the *BbsI* restriction site using primers CFD3apatmerFw and CFD3apatmerRw. This brought the entire gRNA under the ubiquitous U6:3 promoter and created a *BsmBI* restriction site for cloning of annealed primers complementary to target the gene of interest. The plasmid was sequence verified and henceforth referred to as plasmid pCFD3Aptamer. Oligonucleotides used for making the various gRNAs used in this study are listed in Supplementary Table S1.

We further amplified the dCas9 coding sequence (with D10A/H840A substitutions), nuclear localization signal (NLS) sequence and VP64 activation domain from the plasmid lenti dCAS-VP64_Blast (Addgene 61425)^9^, and cloned it into two types of UAS containing plasmids, the pUAST attB vector for S2 cell and somatic tissue specific expression with *EcoRI* and *XhoI* restricition sites^31^, and the pUAS.K10 vector for female germline expression with *KpnI* and *XbaI* restricition sites^32^. We also amplified the DNA region which includes MS2 coat protein (MCP), NLS, p65 activation domain and HSF1 activation domain from the lenti MS2-P65-HSF1_Hygro plasmid (Addgene 61426)^9^, and cloned it into the pUAST and pUAS.K10 vectors using the same restriction enzymes as above. The 5′primers included the Kozak sequence TCAAC in both cases. To modify this system, we cloned dCas9 and NLS without VP64, as well as MCP and NLS without activation domains into the pUAST and pUAS.K10 vectors. For the pUAS.K10 constructs, the reverse primer had a few extra restriction sites which were added after the NLS sequence. The MCP plasmids were further modified by introducing a 3XFLAG tag by cloning annealed oligonucleotides at the *XhoI* site for pUAST-MCP (oligos UAST3xFLAGFw and UAST3xFLAGRw) and at *AscI* for pUAS.K10-MCP (oligos 3xFLAGK10Fw and 3xFLAGK10Rw).

To make CBP fusion constructs we amplified aa 1696–2329 from *Drosophila* CBP (*nejire*), including the bromodomain, PHD domain, and HAT domain, and cloned it into pUAST-MS2-NLS-3XFLAG and pUAS.K10-MS2-NLS-3XFLAG vectors using primers CBPhatUASTFw and CBPhatRw with *Acc65I* and *XbaI* restriction sites for pUAST-MS2-NLS-3XFLAG, and CBPhatK10Fw and CBPhatRw with *AscI* and *XbaI* for pUAS.K10-MS2-NLS-3XFLAG. A F2161A substitution that abolishes CBP HAT activity *in vitro*^17^, was created by amplification from a mutant plasmid^17^ with the same primers and restriction sites. We also fused the CBP wild-type and F2161A HAT domains directly to dCas9 using Cas9.CBPhatUASTFw and CBPhatRw primers and *KpnI* and *XbaI* sites for pUAST-MS2-NLS-3XFLAG, and CBPhatK10Fw and CBPhatRw with *AscI* and *XbaI* for pUAS.K10-MS2-NLS-3XFLAG. All of the resulting plasmids were sequence verified.

We also used the unmodified pCFD3 plasmid for targeting the *twi* and *en* genes in order to compare the effect of dCas9-CBP using gRNAs with or without MS2 loops. The dCas9-VPR activation system (Addgene 78898) was used for comparison, wherein catalytically dead dCas9 (D10A, H39A, H840A, and N863A) is directly fused to the VP64-p65-Rta activation domains under control of the Actin5C promoter^16,33^.

### gRNA selection

We adopted the gRNA targeting sites for *twi, wg, sna, AttC, en* and *hnt* promoters from a published article^33^. For other genomic regions we used the gRNA design software tools http://tools.flycrispr.molbio.wisc.edu/targetFinder/ and http://crispr.mit.edu/. Primers containing homology to the target genes were annealed and cloned into the pCFD3Aptamer plasmid at the *BsmBI* site. gRNAs for *twi* and *en* were also cloned into pCFD3. All the resulting gRNA targeting plasmids were sequence verified.

### Cell culture

*Drosophila* S2 cells were maintained at 25 ^o^C in sterile filtered Schneider’s medium (Gibco) with 10% FBS and 1:100 Penicillin-Streptomycin (10,000U/ml). For seeding, one million cells in 1600 ul of media per well were plated in six well plates for 24 hours before transfection, and plasmid DNA was added to the cells with Effectene Transfection reagent (Qiagen) at a 1:25 ratio. We co-tranfected 250 ng of pAC-GAL4 (Addgene 24344) with 125 ng each of pUAST-dCas9-VP64 or pUAST-dCas9, and pUAST-MCP-p65-HSF1 or pUAST-MCP-CBP or pUAST-MCP-CBPF2161A, and pCFD3Aptamer gRNA plasmids. We used 125 ng each of pAC-GAL4 and pUAST-dCas9-CBP or pUAST-dCas9-CBP F2161A and pCFD3Aptamer or pCFD3 gRNA plasmids. For dCas9-VPR transfections, we used 125 ng each of pAct-dCas9-VPR and pCFD3Aptamer or pCFD3 gRNA plasmids.

Stable cell lines were established by co-transfecting all the components together with a pCoBLast plasmid, and Blasticidin S HCl (10 mg/ml) was added 24 h post transfection. Cells were maintained for one month in Blasticidin media before using them in ChIP-qPCR experiments.

### RT-qPCR

Total RNA was extracted from S2 cells 48 h after transfection with TRIzol LS Reagent (Ambion). The total RNA was purified and concentrated by RNeasy MinElute Cleanup kit (Qiagen). Cleaned RNA was treated with DNaseI (Sigma-Aldrich) and reverse transcribed by the High Capacity RNA-to-cDNA kit (ThermoFisher Scientific) according to the manufacturer’s instructions. The cDNA was used in qPCR with 5X HOT FIREPol Evagreen qPCR Mix Plus (Solis Biodyne) and primers listed in Supplementary Table S1. All transfection experiments were performed at least twice and used two technical qPCR replicates per transfection. The comparative Ct (ΔΔCt) method was utilized to quantify gene expression relative to *RP49* (*RpL32*). One-way ANOVA with post hoc Tukey test was used to calculate statistically significant differences (Supplementary Table S2).

### Western blot

S2 cells were lysed in ice-cold IP buffer (50 mM TRIS pH 8.0, 150 mM NaCl, 1% Triton-X and cOmplete ULTRA protease inhibitor, Roche) 48 hours after transfection for total protein lysate extraction. Cell lysates were centrifuged at 18000 rpm at 4 °C to collect the supernatant and total protein concentration was estimated by Bradford assay. Blots were probed with the primary antibodies anti-Cas9 (Abcam 191468, 1:500), anti-tubulin (Abcam 18251, 1:5000), anti-FLAG (Abcam 1162, 1:4000), anti-acetyl lysine (Cell Signaling Technology # 9441, 1:1000), and HRP coupled secondary anti-mouse (P0260) and anti-rabbit (P0448) antibodies (DAKO/Agilent, both at 1:2000). Amersham ECL Select Western Blotting Detection Reagent (GE Healthcare) was used and signals imaged with a Chemi Doc XRS+ with Image Lab Software (BioRad).

### Transgenic flies and crosses

Flies with gRNAs targeting *twi* and *sog* promoters as well as a *sog* enhancer were generated by injecting pCFD3Aptamer plasmids into the *nos-phiC31; attP40* strain (BL#25709). Transgenic flies were identified by their *vermilion*+ eye phenotype and verified by genomic DNA PCR. They were made homozygous by crossing with second balancer chromosome flies.

To integrate dCas9 fusions, *vas-phiC31; attP-86Fb* flies (BL#24749) were injected with pUAST-dCas9-CBP, pUAST-dCas9-CBPF2161A, pUAS.K10-dCas9-CBP, pUAS.K10-dCas9-CBPF2161A, pUAS.K10-dCas9 or pUAS.K10-dCas9-VP64. The pUAS.K10-MCP-p65-HSF1 plasmid was injected into *vas-phiC31; attP-51D* flies (BL#24483), whereas pUAS.K10-MCP-CBP and pUAS.K10-MCP-CBPF2161A were injected into *nos-phiC31; attP-40 flies* (BL#79604). Transformants were identified by their *white*+ eyes and correct integration confirmed by genomic DNA PCR.

The *alpha-tubulin*-Gal4 (*w*; *matalpha4*-GAL-VP16 V2H) maternal driver was combined with UAS-dCas9 and UAS-MCP fusions. Female progenies from this cross were mated with homozygous pCFD3Aptamer gRNA males and embryos collected.

### *In situ* hybridization

Egg-laying cages with apple juice agar plates were set up to collect and age embryos for 1–5 hours. Embryos were dechorionated in 50% bleach for 3 minutes, fixed and hybridized with digoxygenin-labeled RNA probes as previously described^17^.

### ChIP-qPCR

Chip-qPCR was performed as described previously^34^, with a few modifications. Cells were crosslinked with 1% formaldehyde for 15 minutes at room temperature and the crosslinking reaction was stopped by 125 mM Glycine. Chromatin sonication was done in sonication buffer (50 mM Hepes, 140 mM NaCl, 1 mM EDTA, 1% Triton, 0.1% deoxycholate, 0.5% SDS, 0.5% Sarkosyl and cOmplete ULTRA protease inhibitor, Roche) using Bioruptor plus (Diagenode) at 30 sec ON/OFF at high power for 30 minutes to shear the chromatin at size ranges of 200–500 bp. Immediately after sonication, chromatin material was centrifuged at 18000 rpm for 10 minutes at +4 °C. Supernatant was transferred to new Eppendorf tube and sonication buffer without SDS and sarkosyl was added to dilute their concentration down to 0.1%. The following antibodies H3 (Abcam ab1791) and H3K27ac (Abcam ab4729) were used for immunoprecipitation. The eluted DNA was purified by using ChIP DNA Clean and Concentrator (Zymogen), and used in qPCR to measure the H3K27ac enrichment at the targeted genomic region. All ChIP-qPCR primer sequences are listed in Supplementary Table S1.

## Supplementary information

Supplementary Figures and Tables

Supplementary Table 2

## Data Availability

All data generated or analyzed during this study are included in this published article (and its Supplementary Information files).
