# Supplementary material for: Gene activation by dCas9-CBP and the SAM system differ in target preference
Source: Sci Rep. 2019 Dec 2;9:18104. doi: 10.1038/s41598-019-54179-x (PMC6888908; doi:10.1038/s41598-019-54179-x)
Supplement: Supplementary file 1 — Supplementary Figures and Tables [file 41598_2019_54179_MOESM1_ESM.pdf]

Supplemental Data to

Gene activation by dCas9-CBP and the SAM system differ in target preference

Suresh Sajwan and Mattias Mannervik

Supplemental Figure legends

Figure S1. Time-course of gene activation in *Drosophila* S2 cells. RT-qPCR showing *twi* expression in S2 cells transfected with UAS-dCas9 fusions and *Actin*-Gal4 in the presence of a control gRNA or *twi* promoter gRNA. RNA was harvested at 24, 48, 72 and 96 hours post transfection. Expression is plotted relative to *RP49*, n=2 biological replicates and error bars represent S.E.M.

Figure S2. dCas9-CBP is a better activator than the MCP-CBP fusion plus dCas9 combination. Expression of (**A**) *en* (**B**) *inv* (**C**) *sna* (**D**) *hnt*, and (**E**) *sog* relative to *RP49* in S2 cells transfected with dCas9-CBP or with MCP-CBP plus dCas9 and control gRNA or gRNAs targeting the promoters of these genes as measured by RT-qPCR. n=3 (*en, inv*) or 2 (*sna, hnt, sog*), error bars show S.E.M. Schematic drawings of the loci and location of the gRNAs relative to the transcription start sites (TSS) are shown to the right.

Figure S3. Comparison of gRNAs with and without MS2 loops. S2 cells were transfected with dCas9-CBP or dCas9-VPR and control gRNA or gRNAs with or without MS2 loops (pCFD3) targeting the promoters of *en* (**A**) or *twi* (**B**). Expression is plotted relative to *RP49*, n=2. Error bars represent S.E.M. The gRNAs target identical sequences and only differ by the MS2 loops.

Figure S4. The genes flanking *wg* and *AttC* are not activated by promoter-bound dCas9 fusions. (**A**) *Wnt4* and (**B**) *Wnt6* expression in S2 cells transfected with dCas9 fusions and control or gRNAs targeting the *wg* promoter (same as in Fig. 1A). (**C** and **D**) Expression of *CG43691* and *CG4744* in S2 cells transfected with dCas9 fusions

and control or gRNAs targeting the *AttC* promoter (same as in Fig. 2C). n=3, error bars show S.E.M.

Figure S5. Chromatin state may influence from which genomic positions dCas9-CBP activates transcription. H3K27ac and H3K27me3 ChIP-chip data in S2 cells[21]. The locations of the gRNAs are indicated. The (**A**) *twi* (**B**) *wg* (**C**) *en* (**D**) *AttC* (**E**) *sna* (**F**) *hnt* (**G**) *sog* and (**H**) *Snoo* loci are shown.

Figure S6. Targeting of dCas9-CBP or SAM to *twi*, *sog* or *Snoo* enhancers fails to activate transcription. RT-qPCR showing *twi* (**A**) *CG42741* (**B**) *Fatp2* (**C**) *sog* (**D**) and *Snoo* (**E**) expression in S2 cells transfected with dCas9 fusions and gRNAs that target *twi, sog,* or *Snoo* enhancers. Schematic drawings of the loci and locations of the gRNAs are shown. n=2. Error bars show S.E.M.

Figure S7. No increase in H3K27ac could be detected in dCas9-CBP expressing cells. S2 cells were co-transfected with UAS-dCas9 fusions, Actin-Gal4, gRNA and pCo-Blast plasmids and stable cell lines established by Blasticidin selection. Four cell lines (expressing dCas9 control, dCas9-CBP, dCas9-CBP F2161A, or SAM) for each gRNA were generated. ChIP-qPCR used H3 and H3K27ac antibodies. Average values from several amplicons along the *twi* (4 promoter and 3 exon amplicons), *wg* (3 enhancer and 2 promoter amplicons) and *RP49* loci are plotted as fold over an intergenic control region, and further normalized to H3 occupancy. H3K27ac enrichment at the (**A**) *twi* (**B**) *RP49* (**C**) *wg* and (**D**) *RP49* loci in the different cell lines is shown. The *RP49* promoter has high levels of H3K27ac and is used as a positive control. Error bars show S.E.M., n=2.

Figure S8. Expression of the CBP HAT-domain results in a global increase in protein acetylation. Western blot showing protein acetylation. Protein extract from untreated S2 cells or S2 cells transfected with dCas9- or MCP-fusion proteins were probed with an anti-acetyl lysine antibody (A) and tubulin as a control (B). Expression of dCas9-CBP and MCP-CBP results in a strong increase in acetylation of multiple proteins, whereas the corresponding proteins with a F2161A mutation that disrupts catalytic activity do not.

Figure S9. Uncropped images of Western blot shown in Fig. 1C.

Table S1. List of primers.

Table S2. Statistical analysis and fold activation

Table S3. Comparison of histone acetylation in S2 cells and early embryos.

Figure S1

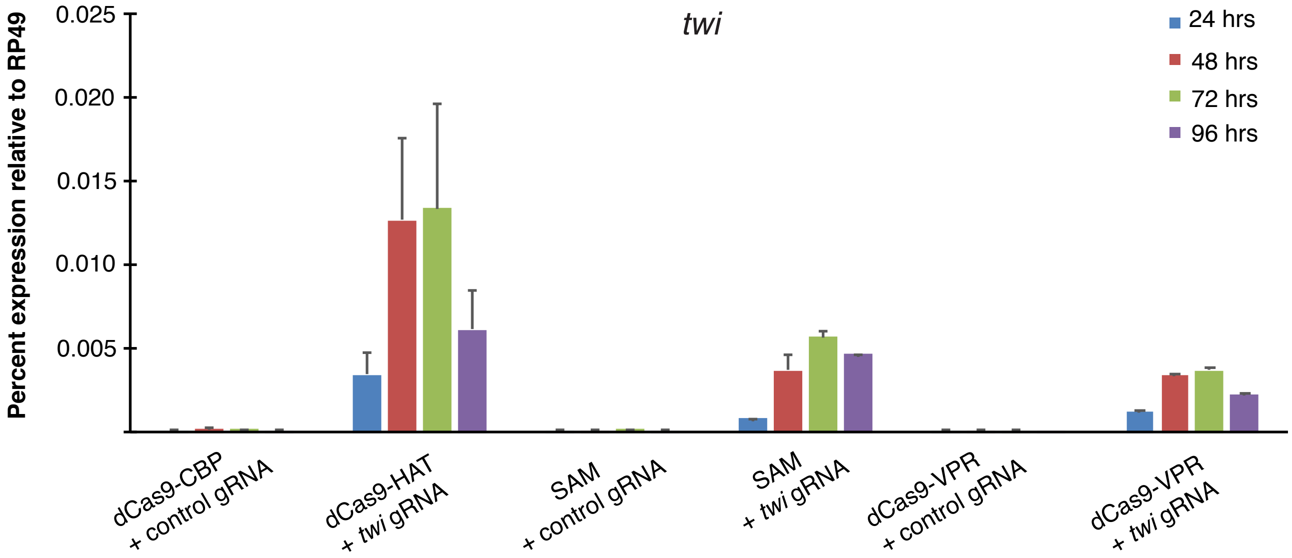

twi
Percent expression relative to RP49
0.025
0.020
0.015
0.010
0.005
24 hrs
48 hrs
72 hrs
96 hrs
dCas9-CBP + control gRNA
dCas9-HAT + twi gRNA
SAM + control gRNA
SAM + twi gRNA
dCas9-VPR + control gRNA
dCas9-VPR + twi gRNA

Figure S2

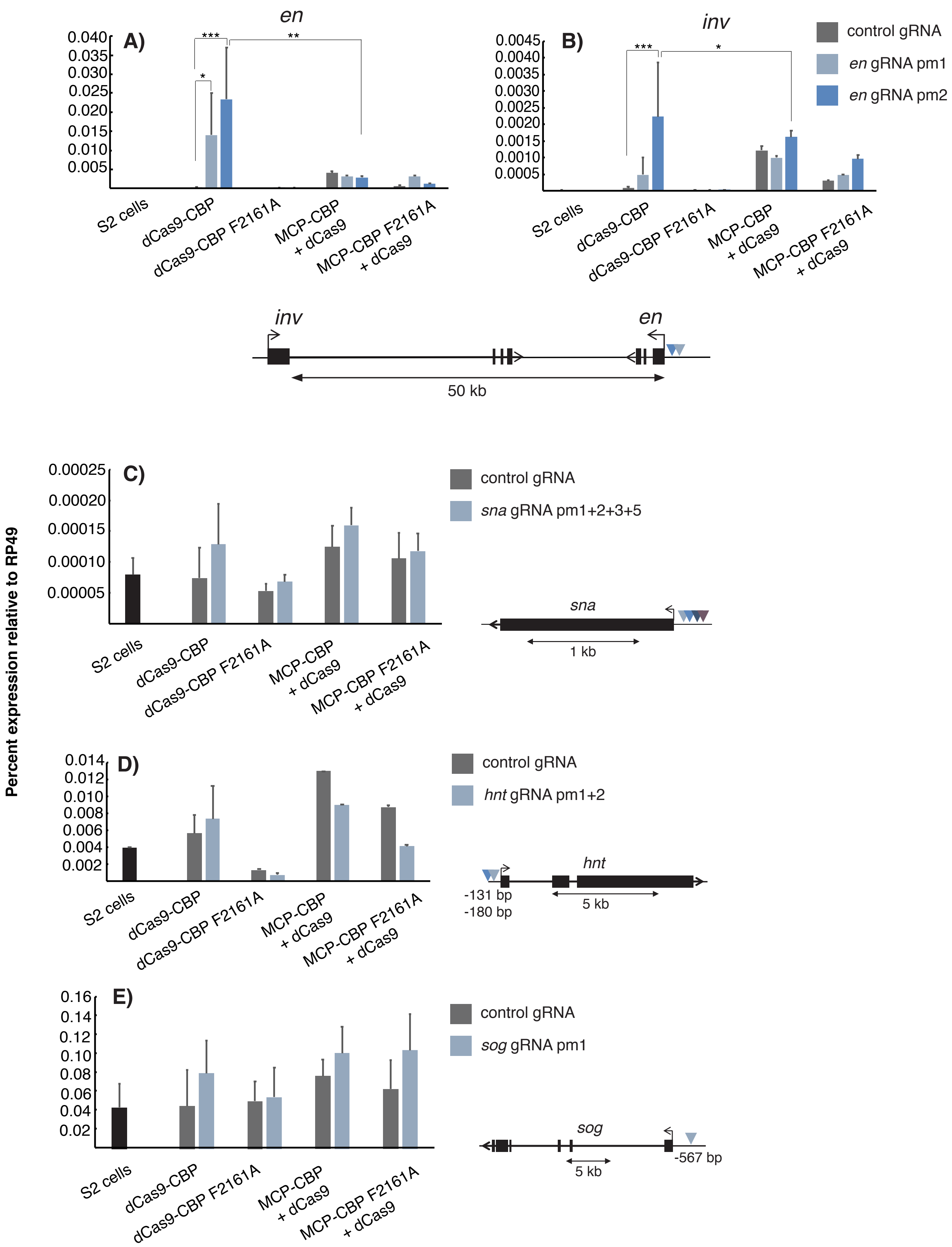
Percent expression relative to RP49
A)
en
0.040
0.035
0.030
0.025
0.020
0.015
0.010
0.005
***
**
*
S2 cells
dCas9-CBP
dCas9-CBP F2161A
MCP-CBP + dCas9
MCP-CBP F2161A + dCas9
B)
inv
0.0045
0.0040
0.0035
0.0030
0.0025
0.0020
0.0015
0.0010
0.0005
***
*
control gRNA
en gRNA pm1
en gRNA pm2
inv
en
50 kb
C)
0.00025
0.00020
0.00015
0.00010
0.00005
control gRNA
sna gRNA pm1+2+3+5
sna
1 kb
D)
0.014
0.012
0.010
0.008
0.006
0.004
0.002
control gRNA
hnt gRNA pm1+2
hnt
-131 bp
-180 bp
5 kb
E)
0.16
0.14
0.12
0.10
0.08
0.06
0.04
0.02
control gRNA
sog gRNA pm1
sog
5 kb
-567 bp

Figure S3

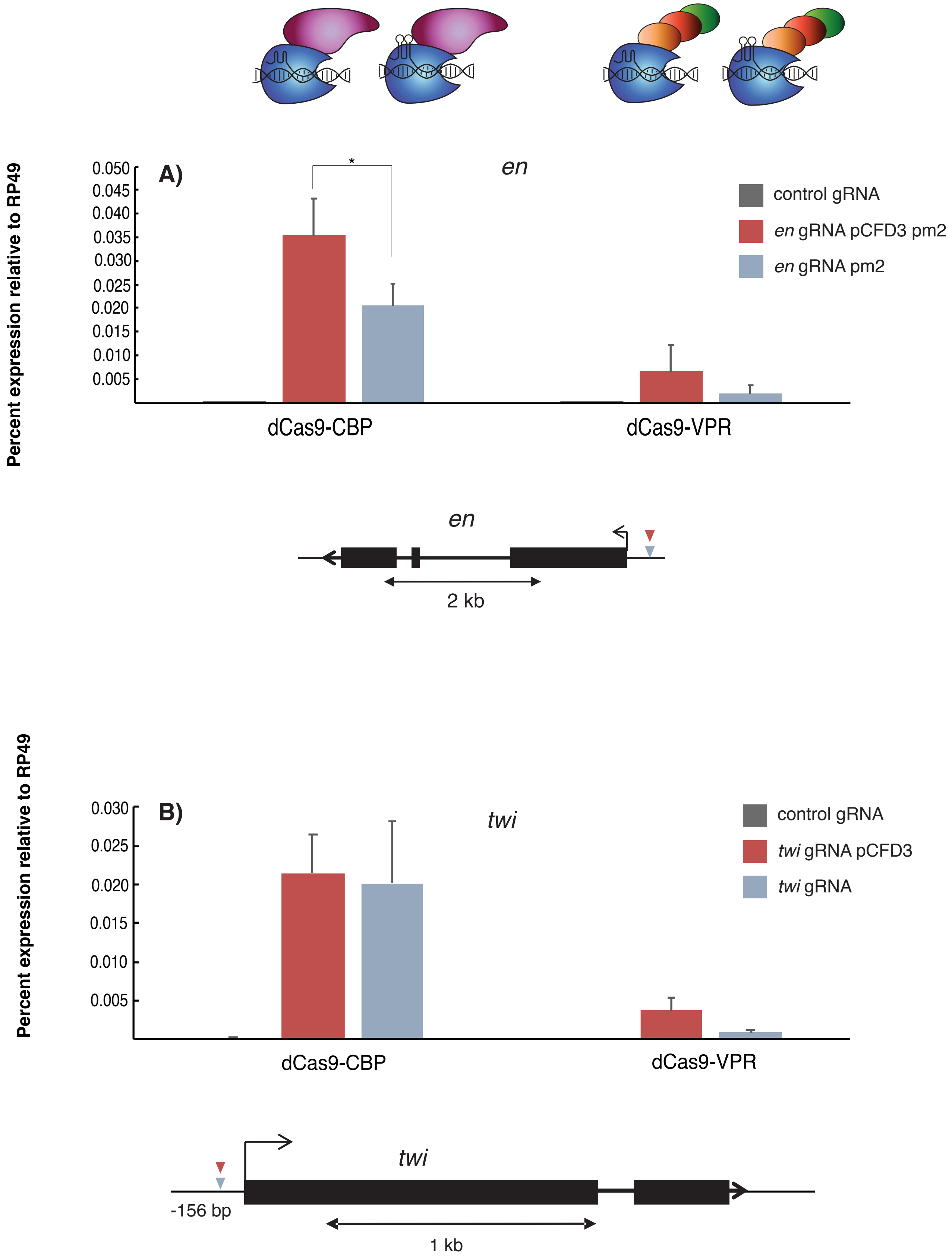

A)
en
Percent expression relative to RP49
0.050
0.045
0.040
0.035
0.030
0.025
0.020
0.015
0.010
0.005
*
control gRNA
en gRNA pCFD3 pm2
en gRNA pm2
dCas9-CBP
dCas9-VPR
en
2 kb
B)
twi
Percent expression relative to RP49
0.030
0.025
0.020
0.015
0.010
0.005
control gRNA
twi gRNA pCFD3
twi gRNA
dCas9-CBP
dCas9-VPR
twi
-156 bp
1 kb

Figure S4

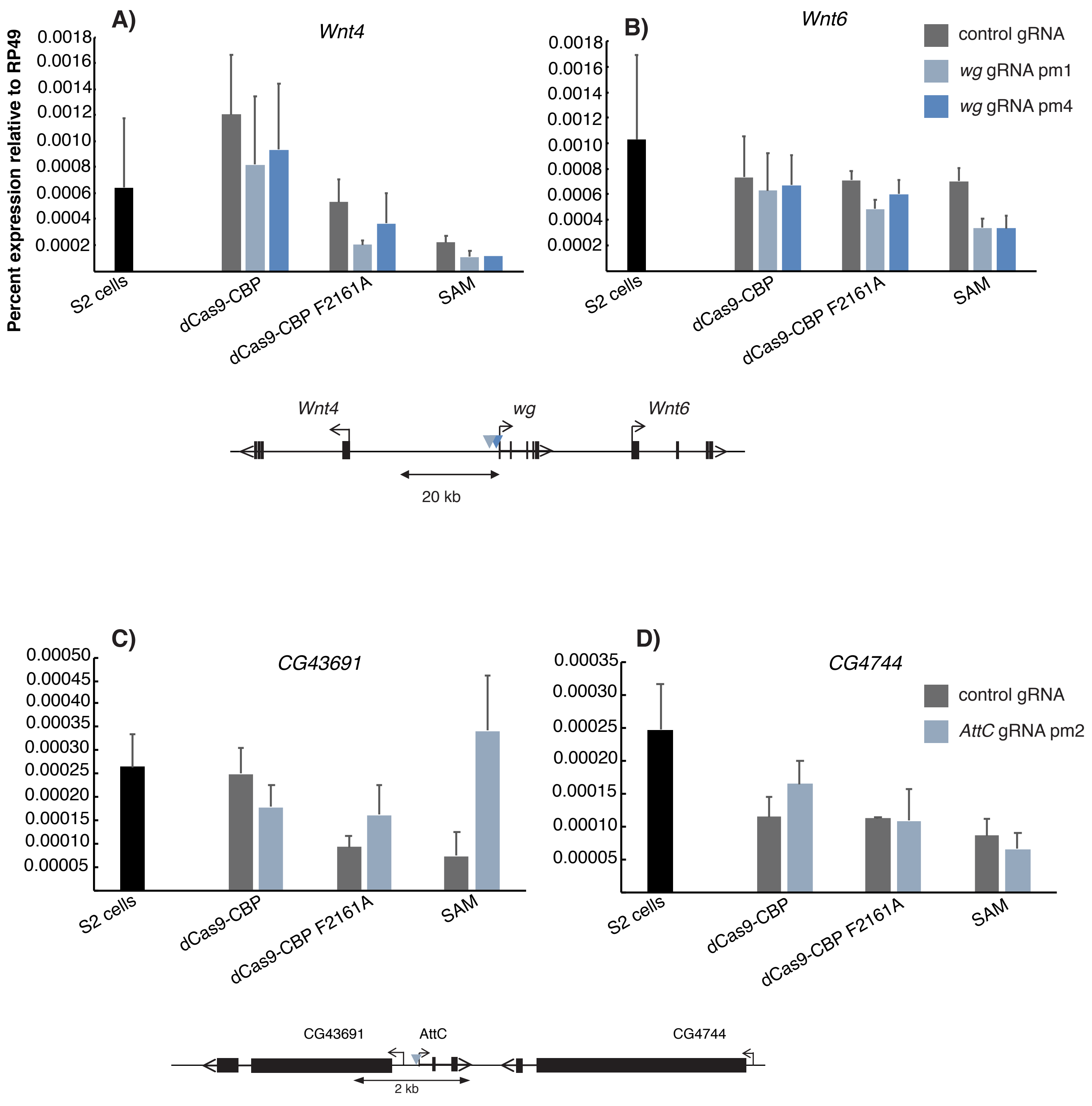
A)
Wnt4
Percent expression relative to RP49
0.0018
0.0016
0.0014
0.0012
0.0010
0.0008
0.0006
0.0004
0.0002
S2 cells
dCas9-CBP
dCas9-CBP F2161A
SAM
B)
Wnt6
control gRNA
wg gRNA pm1
wg gRNA pm4
Wnt4
wg
Wnt6
20 kb
C)
CG43691
0.00050
0.00045
0.00040
0.00035
0.00030
0.00025
0.00020
0.00015
0.00010
0.00005
D)
CG4744
control gRNA
AttC gRNA pm2
CG43691
AttC
CG4744
2 kb

Figure S5

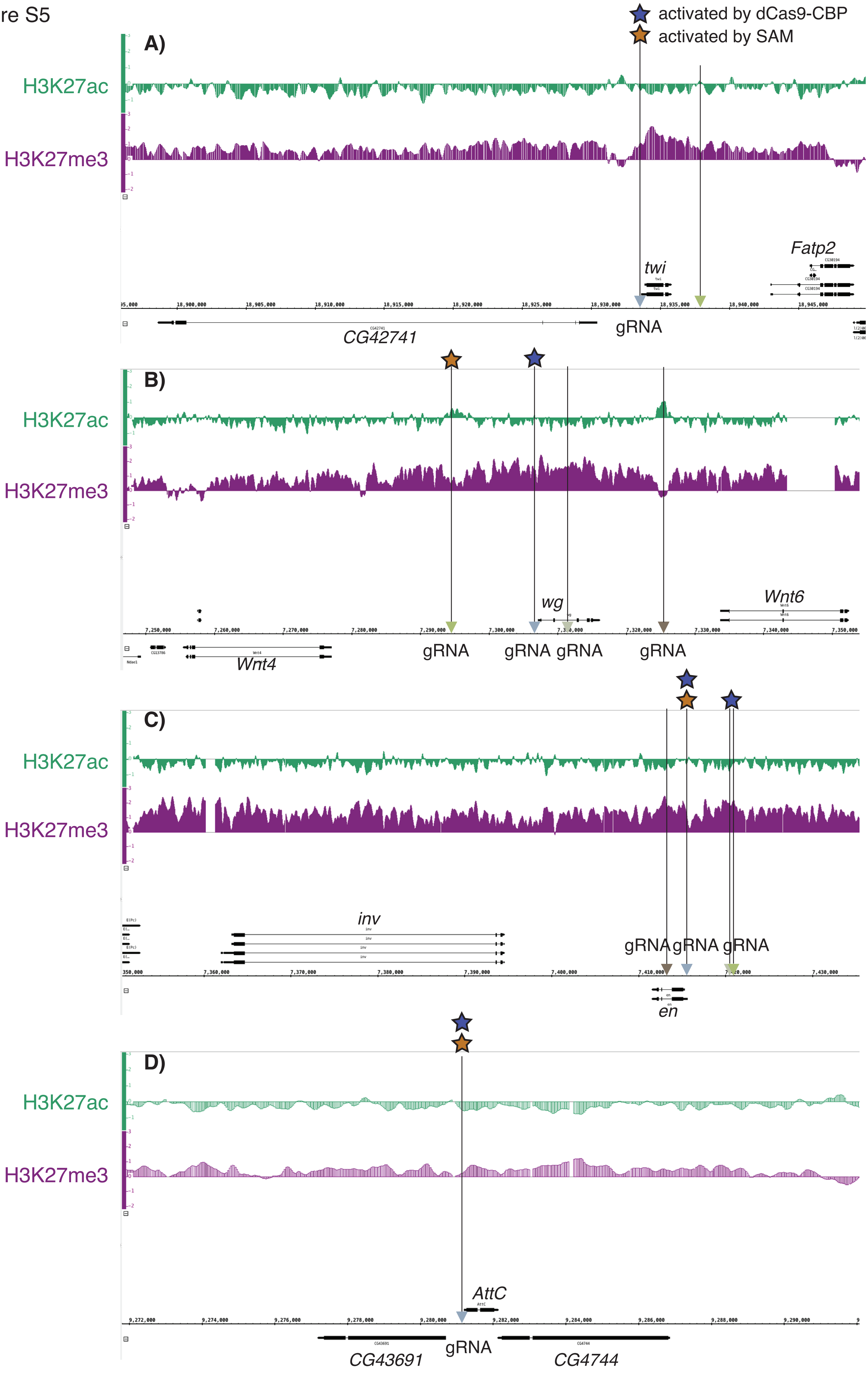

activated by dCas9-CBP
activated by SAM
A)
H3K27ac
H3K27me3
twi
Fatp2
CG42741
gRNA
B)
H3K27ac
H3K27me3
wg
Wnt6
Wnt4
gRNA
gRNA
gRNA
gRNA
C)
H3K27ac
H3K27me3
inv
gRNA
gRNA
gRNA
en
D)
H3K27ac
H3K27me3
AttC
gRNA
CG43691
CG4744

Figure S5 continued

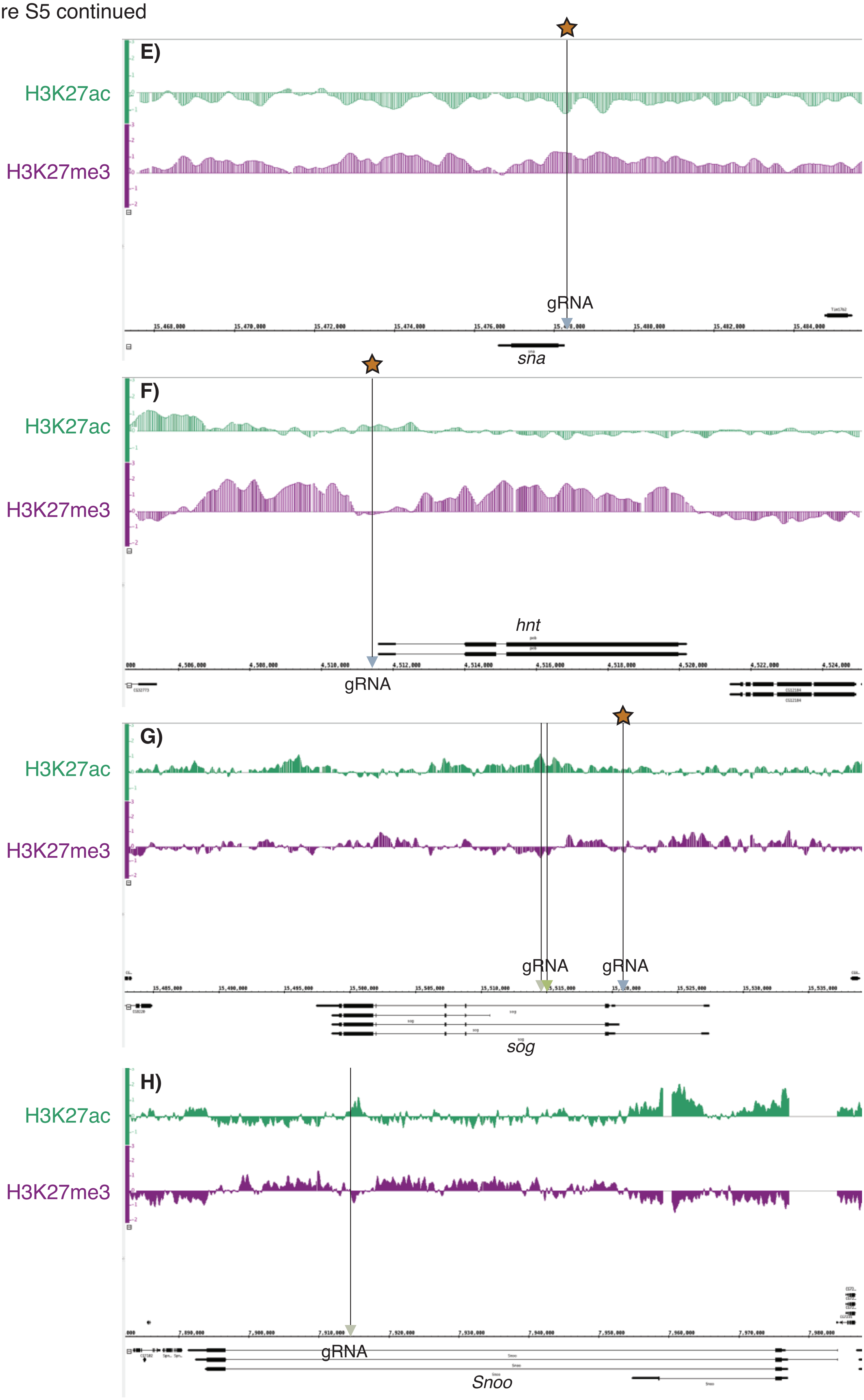

Figure S6

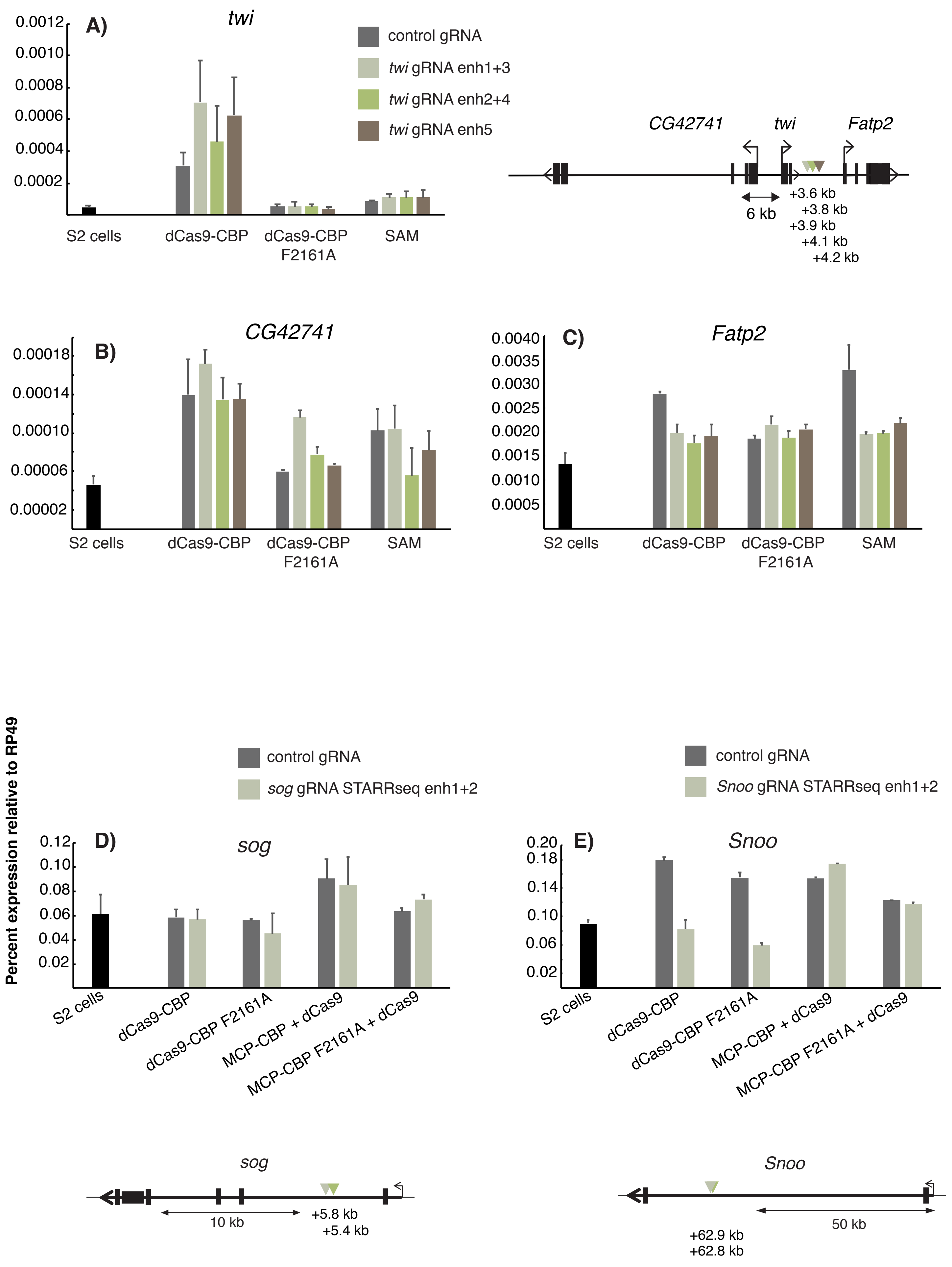
A)
twi
0.0012
0.0010
0.0008
0.0006
0.0004
0.0002
control gRNA
twi gRNA enh1+3
twi gRNA enh2+4
twi gRNA enh5
S2 cells
dCas9-CBP
dCas9-CBP F2161A
SAM
CG42741
twi
Fatp2
6 kb
+3.6 kb
+3.8 kb
+3.9 kb
+4.1 kb
+4.2 kb
B)
CG42741
0.00018
0.00014
0.00010
0.00006
0.00002
C)
Fatp2
0.0040
0.0035
0.0030
0.0025
0.0020
0.0015
0.0010
0.0005
Percent expression relative to RP49
control gRNA
sog gRNA STARRseq enh1+2
Snoo gRNA STARRseq enh1+2
D)
sog
0.12
0.10
0.08
0.06
0.04
0.02
E)
Snoo
0.20
0.18
0.14
0.10
0.06
0.02
S2 cells
dCas9-CBP
dCas9-CBP F2161A
MCP-CBP + dCas9
MCP-CBP F2161A + dCas9
10 kb
+5.8 kb
+5.4 kb
50 kb
+62.9 kb
+62.8 kb

Figure S7

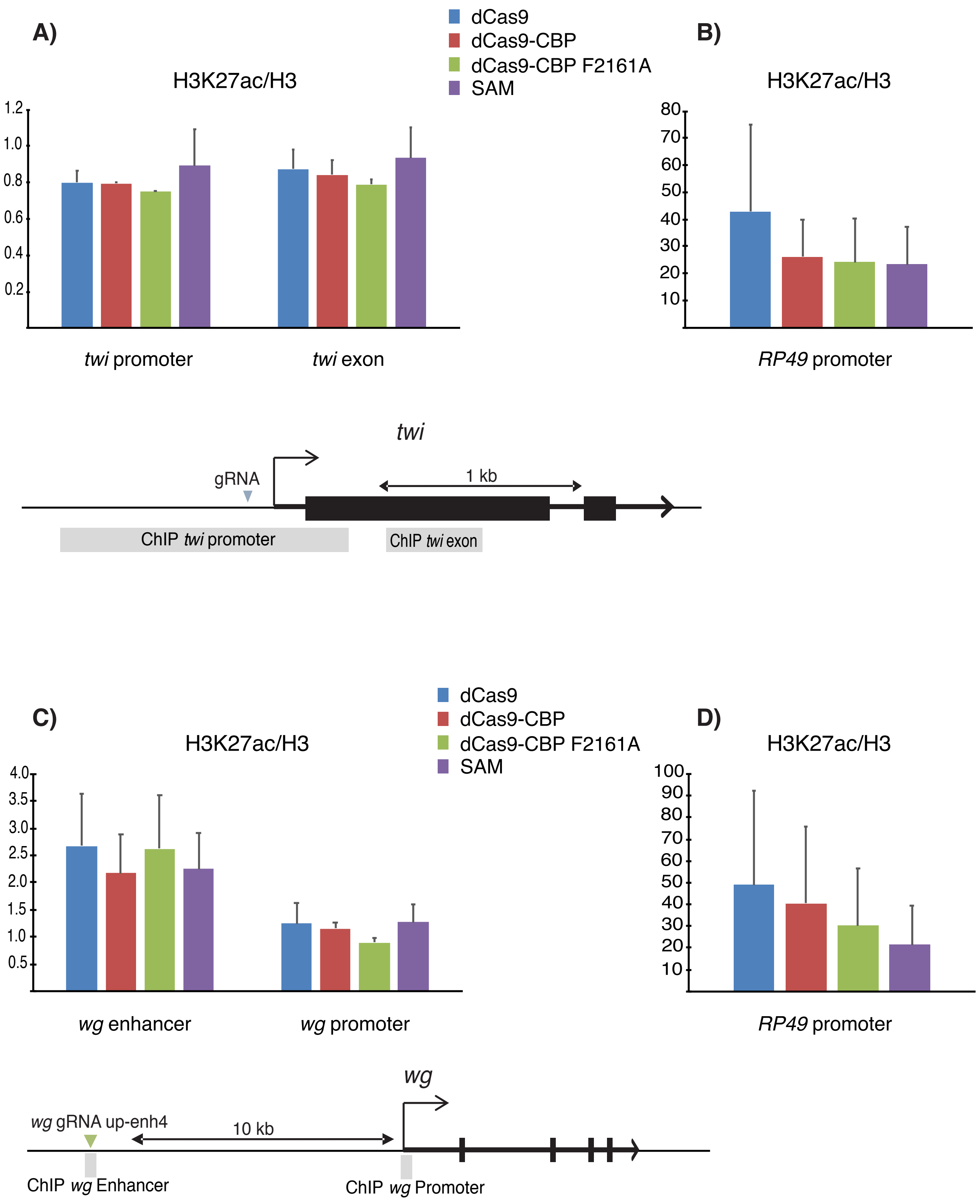
A)
H3K27ac/H3
1.2
1.0
0.8
0.6
0.4
0.2
twi promoter
twi exon
dCas9
dCas9-CBP
dCas9-CBP F2161A
SAM
B)
H3K27ac/H3
80
70
60
50
40
30
20
10
RP49 promoter
twi
gRNA
1 kb
ChIP twi promoter
ChIP twi exon
C)
H3K27ac/H3
4.0
3.5
3.0
2.5
2.0
1.5
1.0
0.5
wg enhancer
wg promoter
dCas9
dCas9-CBP
dCas9-CBP F2161A
SAM
D)
H3K27ac/H3
100
90
80
70
60
50
40
30
20
10
RP49 promoter
wg
wg gRNA up-enh4
10 kb
ChIP wg Enhancer
ChIP wg Promoter

Figure S8

A)

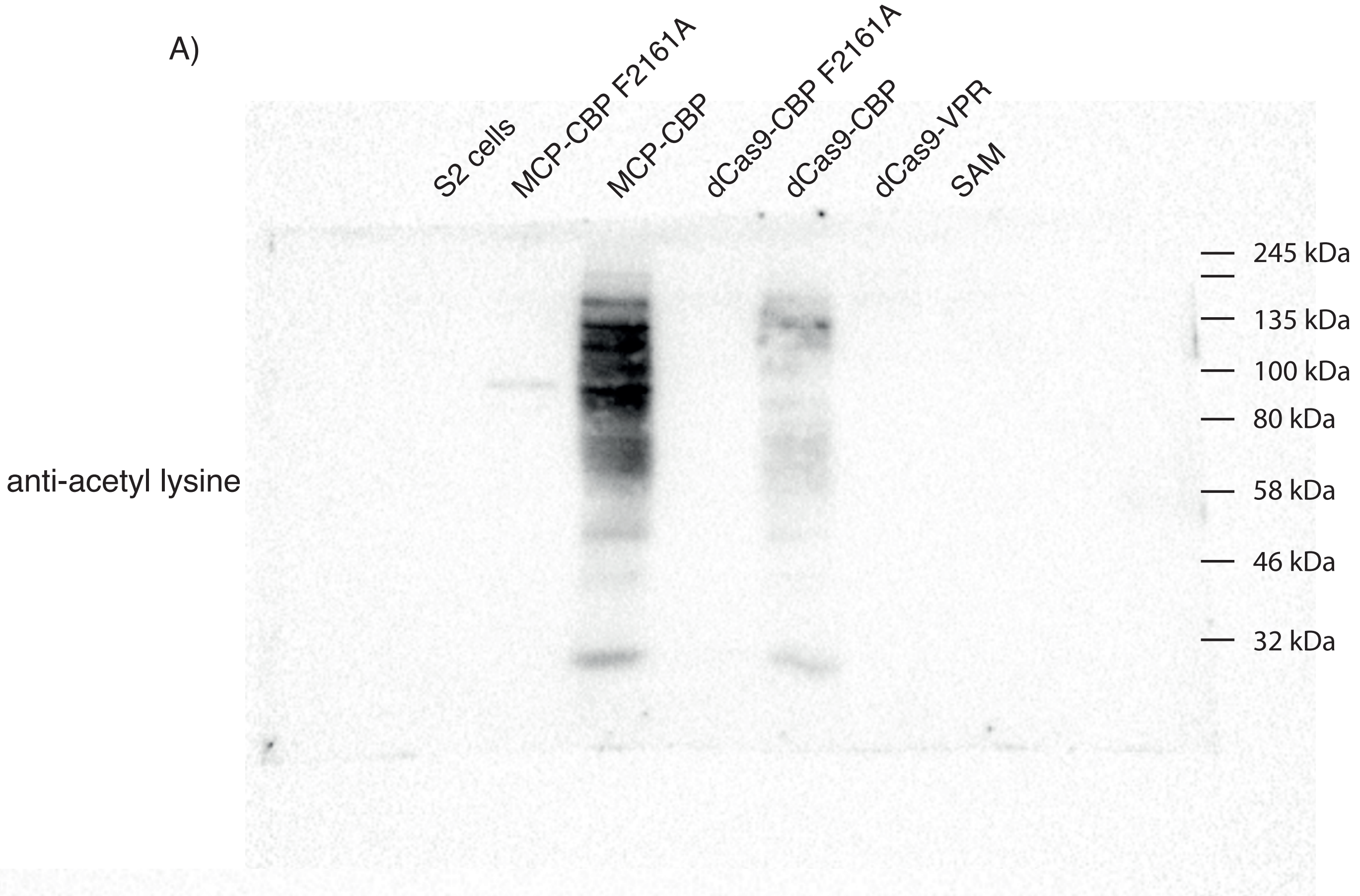

S2 cells
MCP-CBP F2161A
MCP-CBP
dCas9-CBP F2161A
dCas9-CBP
dCas9-VPR
SAM
245 kDa
135 kDa
100 kDa
80 kDa
58 kDa
46 kDa
32 kDa
anti-acetyl lysine

B)

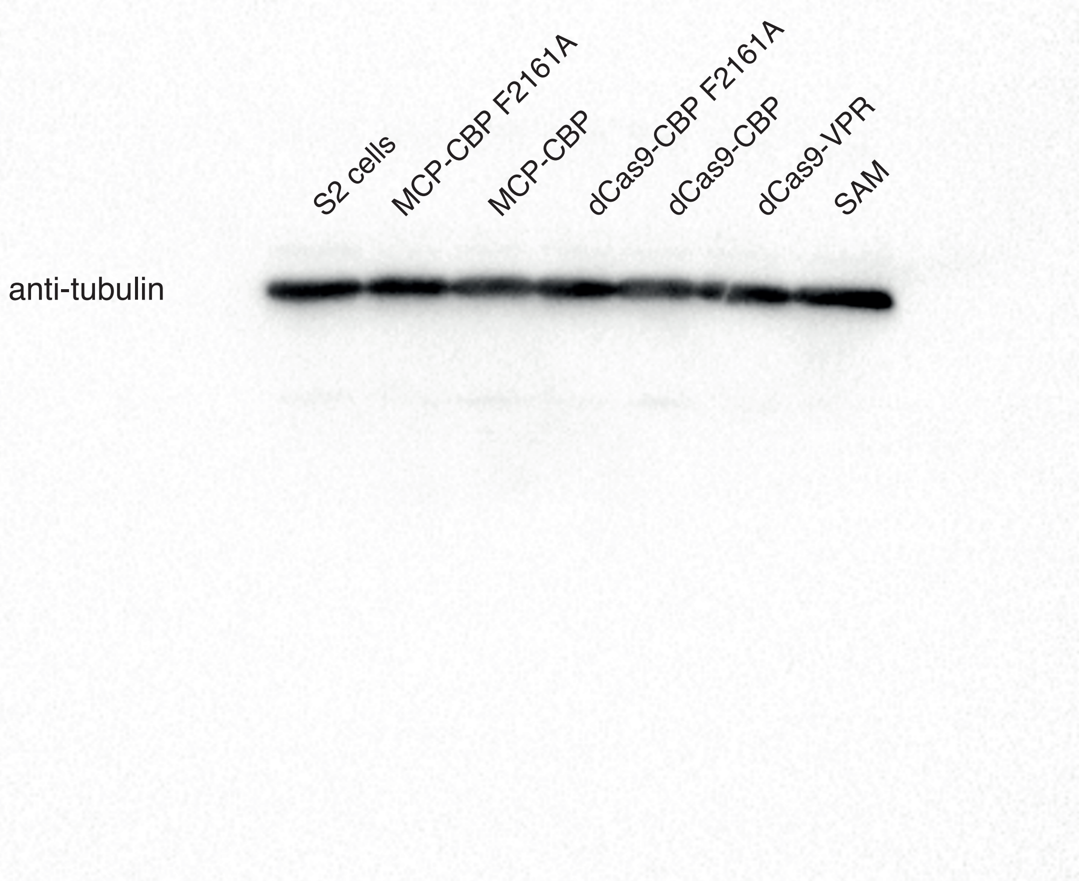

S2 cells
MCP-CBP F2161A
MCP-CBP
dCas9-CBP F2161A
dCas9-CBP
dCas9-VPR
SAM
anti-tubulin

Figure S9

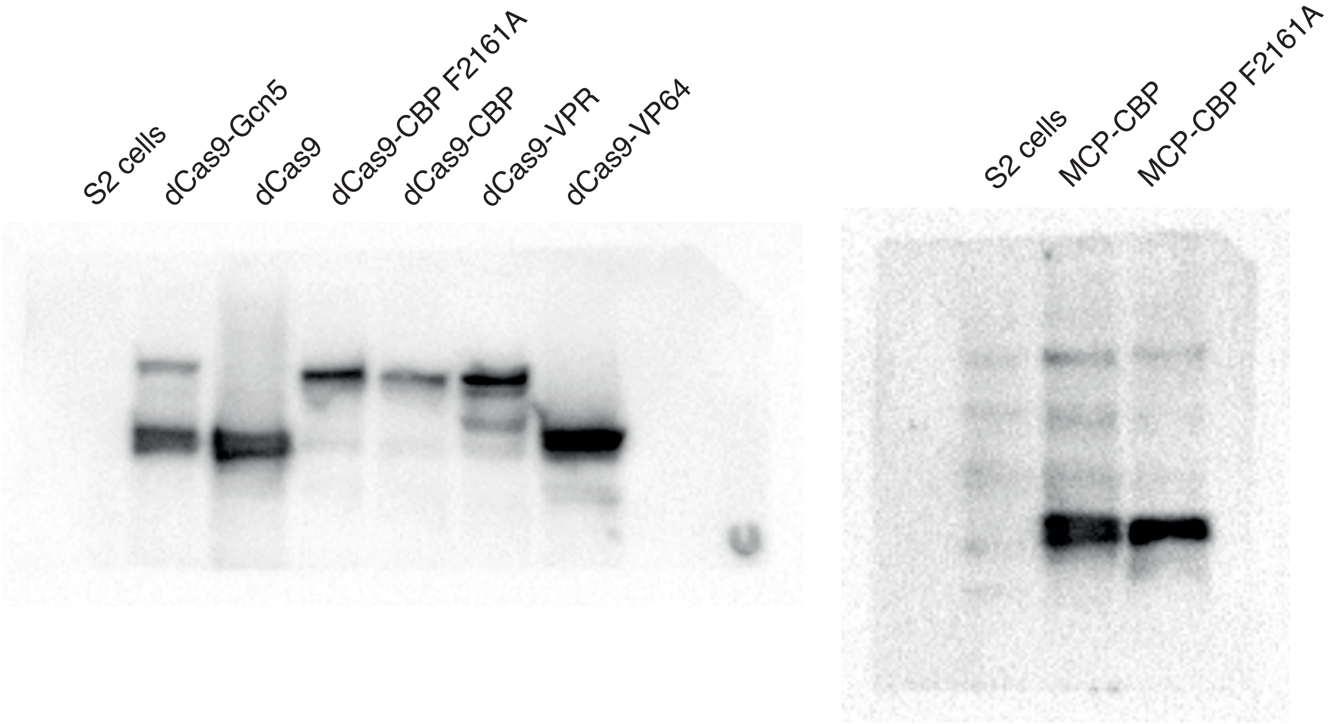
S2 cells
dCas9-Gcn5
dCas9
dCas9-CBP F2161A
dCas9-CBP
dCas9-VPR
dCas9-VP64
S2 cells
MCP-CBP
MCP-CBP F2161A

Table S1. List of oligonucleotides

List of gene promoters targeted and the gRNA sites used

gRNAs used for targeting gene promoter	sgRNA with PAM underlined	Position relative to TSS	Reference
twist	CCTGACGTCATACCTGCCGATGC	minus 156 bp	Reference 22
wgPmFw1	ATGAGGTTGCGCAAATAATCGGG	minus 363bp	Reference 22
wgPmFw4	GCAGCTGCAATGCAGGAGTCAGG	minus 145bp	Reference 22
engrlpmFw1	AACTGTCACGGTGGAAAGAGAGG	minus 78 bp	Reference 22
engrlpmFw2	GCGTTAACTCTCCCCGACGTCGG	minus 20bp	Reference 22
AttCPmFw2	GTGAACCACCTGGTCATTCGGGG	minus 140bp	Reference 22
snailpmfw1	CCGACGCCGCTGTCGCCATTTGG	minus 68bp	Reference 22
snailpmfw2	AAAGTGCTGTTGTTGTTGCTAGG	minus 117bp	Reference 22
snailpmfw3	GAAATACGCAATAAGGGTATGGG	minus 141bp	Reference 22
snailpmfw5	TCCATTTCCCACCTCTCTCTCGG	minus 235bp	Reference 22
hntpmFw1	GAGAGAAGAGAGAAGCAGTCTGG	minus 131bp	Reference 22
hntpmFw2	ATTTGAAACGAAGAATGAGAAGG	minus 180bp	Reference 22
sogPmFw1	GAAGAGCGCGAGAGATAGAGAGG	minus 567 bp	This study
sogPmFw2	CATGCAACGGCAACATGCAGCGG	minus 45 bp	This study
sogPmFw3	CAGCAGCAACATCTAACGATCGG	minus 88 bp	This study
sogPmFw4	GATTACGTCTTGTTACATTTTGG	minus 398 bp	This study
quas	CTCGGGTAATCGCTTATCCTCGG		Reference 22

Oligonucleotides for cloning into the pCFD3Aptamer plasmid are based on the sequences above with additional nucleotides that generate sticky overhangs after annealing (lowercase letters) for efficient ligation.

e.g. twist gRNA GCATCGGCAGGTATGACGTC AGG

TwistFw **5- gtcg**GCATCGGCAGGTATGACGTC -3

TwistRw 5- **aaac**GACGTCATACCTGCCGATGC -3

List of enhancers targeted and the gRNA sites used

**gRNAs used for targeting gene enhancers**	**sgRNA with PAM underlined**	**Position relative to TSS**
en.En.Fw2	AGCCACGCTAACGCAACAGGCGG	minus 4628 bp
en.En.Fw3	GACCAGCAGCAAAACCTTCTGGG	minus 4894 bp
en.En.Fw4	CTTTCATGGGTTCGTAGGTAAGG	minus 5435 bp
en.En.Fw5	GTTCCGACGTGACCCGAGCTCGG	minus 5697 bp
en.FstExon.Fw1	GCGGGGGAAGCCCCTTCGAAAGG	plus 2088 bp
en.FstExon.Fw2	CTTGGGGAGCGTTATGTTGGCGG	plus 1705 bp
wgEnFw1	CAGGGGCGTGACACAGTCATTGG	plus 4425 bp
wgEnFw2	CATGCGCCGACGTCTTATCGCGG	plus 4518bp
wgEnFw3	CGGGAATTGGAGGCTCACTTCGG	plus 4661 bp
wgEnFw4	AGCAAACGACTAAAAGGCTCTGG	minus 12266 bp
wgEnFw5	TCAAGTCCAGATATCTCGCCTGG	minus 12148bp
wgEnFw6	GCTGTAACTAATTGGCGCTCTGG	plus 18635 bp
wgEnFw7	TGATAAGATCAGAAATACCATGG	plus 18389 bp
twi.EnFw1	GCAAAAAGGACCTCATGCCAAGG	plus 3636 bp
twi.EnFw2	AGGTCCCTGATCCTCCGTCCTGG	plus 3783 bp
twi.EnFw3	GGAAGAGCGGCTCAAATGACAGG	plus 3881 bp
twi.EnFw4	AGTGGAGAACTAAAGTGAGTTGG	plus 4055 bp
twi.EnFw5	TACTCTGTAATTGCAGTTACTGG	plus 4162 bp
sogEnFw1	GCGGGATTAGAGGTGCGAGCAGG	plus 1570 bp
sogEnFw2	GGCTATATGGCTGTATGGTGCGG	plus 1498 bp

**gRNAs used for targeting STARR-seq enhancers**	**sgRNA with PAM underlined**	**Position relative to TSS**
sog fw1	GATTTGGTTTGTAAATGGGAAGG	plus 5758 bp
sog fw2	GTCACGCGACACGCGATTTGCGG	plus 5426 bp
snoo fw1	CTAAGGAAGTGGATGAAGATCGG	plus 62902 bp
snoo fw2	AGTTGCATGTGTATGAGATGTGG	plus 62834 bp

Primers used for RT-qPCR

**Genes**	**Forward Primer**	**Reverse Primer**	**Reference**
twist	AAGTCCCTGCAGCAGATCAT	CGGCACAGGAAGTCAATGTA	Reference 22
wingless	CCAAGTCGAGGGCAAACAGAA	TGGATCGCTGGGTCCATGTA	Reference 22
engrailed	TCCGTGATCGGTGACATGAGT	CGCCGACGTATCATCCACATC	Reference 22
Attacin-C	CGCCACCCAGAATCTACAGG	CTTAGGTCCAATCGGGCATCG	Reference 22
snail	GTCGGTCTACAGCTACCAGC	GCAGTGGGATGTCATTTCGC	Reference 22
hindsight	ACATCCGGTGCCACAATTA	AGGGATGAAGCCGAGGATAGC	Reference 22
sog	CCAGTTCGCCGGCATAGAGC	CCCGGGATAGGCCCAACATC	This study
CG42741	GCCCTCCGGTTCAGGTTGA	TGTTGGCCGACGAGAATTTG	This study
Fatp2 (CG44252)	CGCCTCCGGTTACTTTTCAGAC	ATGGCGTGGCCAGGATGTAG	This study
invected	GAGGACAAAAGGCCGCGAAC	TCCGTCAGATAGCGATTCTCG	This study
Wnt4	ACAGGTGTCATCGAGTCGC	AGGGCGAGGTCTCCAAATAGT	This study
Wnt6	CAAGCAAAGGCAAAGGCGTA	CCGTGACACTTGCACTCCAG	This study
CG43691	TCGCTACACAACATTAGGAGCA	TCTTCTTGTGGACCAACTGC	This study
CG4744	TACAAAAATCGTCGGGAAAGTGC	GGAGATTTTGCCCGTGATTAGC	This study
Snoo	CGATGTGGAGAATCGGGAGA	CATGAGCATTTCCGCCTTTCTT	This study
Tim17b2	CCACAGGGACTAGGACGCA	ATGCTGAACACTGCACCCC	This study
CG4161	TCTGATGCTCAGCGAGATGG	TTCCCCACAGGTTAATCCGC	This study
Worniu	AGCGTCCGATAATGGTGGA	TTAGCCATCCAATGGGGCTC	This study
Rp49	ATCGGTTACGGATCGAACAA	GACAATCTCCTTGCGCTTCT	Reference 22

Primers used for Chip-qPCR

**Genes**	**Region**	**SubRegion**	**Forward Primer**	**Reverse Primer**
twist	Promoter	Twist_CBP	CATTCGCCTGCGATTTTCTCG	CATCAGTTAGTTGCCAACCATCGTAAA
		TwiChip1	CAATTTGAGCAATGGCCGGA	TCCGTAGATATTCTTCACGCCC
		TwiChip5	TGCTGCTGGACATAAGCTACA	CAGTGGGGCGTACTGGG
		TwiChip6	TTCCGCCATTGAGTGGGAG	CTTACGGAACGCAACTGACG
	Gene body	ChipTwiexon 1	GCAACAATCCGAGTGGCTTC	GGTTTGGCAATCGTAGTCGC
		ChipTwiexon 2	GATTACTTGCCCACCACCGT	AGCTCCTCGTCGGGAAAAT
		ChipTwiexon 3	GATGCACTTCCAAAACGCCT	CCATGTCATCCCGATCCGAG
wingless	Enhancer	WgEnChip1	CAGCCAAGCGAGTCCAACTA	AAGGAGGAGCAAGGCAGATG
		WgEnChip2	TTTATGCCCTCCTCACTCGG	GTTCGACCAATGCGGAACAG
		WgEnChip3	GCAATACAGTGGAGCCTCGT	TGGTTGTCAACTGACCTGTGT
	Promoter	WgPmChip1	TGCGATCCGAAGACGAAGAC	AGATTGTTGCCCGGTTCAGT
		WgPmChip2	CGCACCGTGTGTTTCAGTTA	ACACTCGGCTCGCTCTAGTT
Rpl32	Promoter		TTCACGATCTTGGGCCTGTATG	TTGTTGTGTCCTTCCAGCTTCA
Ig1c	Intergenic		AGCGTTGTGCGAAGGTAAAC	ATGGCCATTCCCCAAGGTCAT

Primers used for constructing pCFD3Aptamer plasmid. Restriction sites used are bold and italicized.

Plasmids	Primers name	Primers sequence
pCFD3Aptamer	CFD3apatmerFw	ATGCT***GAAGAC***AAGTCGGGAGACGGGATACCGTCTCTG
	CFD3apatmerRw	CGCAG***GAAGAC***AGAAACAAAAAAAGCACCGACTCGGTG

Primers used for constructing pUAST based plasmids. Restriction sites used are bold and italicized.

Plasmids	Primers name	Primers sequence
pUAST-dCas9.VP64	UASTCas9Fw	AATTGG***GAATTC***AACATGAAAAGGCCGGCGGCCACG
	UASTCas9VP6Rw	GGTACC***CTCGAG***GTTAATCAGCATGTCCAGGTCG
pUAST-dCas9	UASTCas9Fw	AATTGG***GAATTC***AACATGAAAAGGCCGGCGGCCACG
	UASTCas9Rw	GGTACC***CTCGAG***AGCGGCCGCCACCTTCCTCT
pUAST-MCP.p65. HSF1	UASTMS2Fw	AATTGG***GAATTC***AACATGGCTTCAAACTTTACTCAG
	UASTMS2P65Rw	GGTACC***CTCGAG***GGAGACAGTGGGGTCCTTGG
pUAST-MCP	UASTMS2Fw	AATTGG***GAATTC***AACATGGCTTCAAACTTTACTCAG
	UASTCas9Rw	GGTACC***CTCGAG***AGCGGCCGCCACCTTCCTCT
pUAST-MS2.NLS. 3XFLAG	UAST3xFLAGFw	TCGAGGACTACAAAGACCATGACGGTGATTATAAAGATCATGACATCGACTACAAAGACGATGACGACAAG
	UAST3xFLAGRw	TCGACTTGTCGTCATCGTCTTTGTAGTCGATGTCATGATCTTTATAATCACCGTCATGGTCTTTGTAGTCC
pUAST-MS2.NLS. 3XFLAG. HAT	CBPhatUASTFw	TATT***GGTACCA***CAATTCAATCCCGAGGAACTGCG
	CBPhatRw	ATCT***TCTAGA***TCAGTCCTGACCCTGGTTATGCAG
pUAST-MS2.NLS. 3XFLAG. HAT F2161A	CBPhatUASTFw	TATT***GGTACCA***CAATTCAATCCCGAGGAACTGCG
	CBPhatRw	ATCT***TCTAGA***TCAGTCCTGACCCTGGTTATGCAG
pUAST-dCas9.NLS.HAT	Cas9.CBPhatUAST.Fw	TATT***GGTACC***CAATTCAATCCCGAGGAACTGCG
	CBPhatRw	ATCT***TCTAGA***TCAGTCCTGACCCTGGTTATGCAG
pUAST-dCas9.NLS.HAT F2161A	Cas9.CBPhatUAST.Fw	TATT***GGTACC***CAATTCAATCCCGAGGAACTGCG
	CBPhatRw	ATCT***TCTAGA***TCAGTCCTGACCCTGGTTATGCAG

Primers used for constructing pUAS.K10 based plasmids. Restriction sites used are bold and italicized.

Plasmids	Primers name	Primers sequence
pUAS.K10-dCas9.VP64	K10Cas9Fw	AGTATG***GGTACC***AACATGAAAAGGCCGGCGGCCACG
	K10Cas9VP6Rw	AGCAAG***TCTAGA***GAATTCGGCGCGCCGTTAATCAGCATGTCCAGGTC
pUAS.K10-dCas9	K10Cas9Fw	AGTATG***GGTACC***AACATGAAAAGGCCGGCGGCCACG
	K10Cas9Rw	GTCGAC***TCTAGA***GAATTCGGCGCGCCAGCGGCCGCCACCTTCCTCTTTTTC
pUAS.K10-MCP.p65.HSF1	K10MS2Fw	TTCATT***GGTACC***AACATGGCTTCAAACTTTACTCAGTTCG
	K10MS2P65Rw	GTCGAC***TCTAGA***GAATTCGGCGCGCCGGAGACAGTGGGGTCCTTGGCTTTGG
pUAS.K10-MCP	K10MS2Fw	TTCATT***GGTACC***AACATGGCTTCAAACTTTACTCAGTTCG
	K10Cas9Rw	GTCGAC***TCTAGA***GAATTCGGCGCGCCAGCGGCCGCCACCTTCCTCTTTTTC
pUAS.K10-MS2.NLS.3XFLAG	3xFLAGK10Fw	CGCGGACTACAAAGACCATGACGGTGATTATAAAGATCATGACATCGACTACAAAGACGATGACGACAAGGG
	3xFLAGK10Rw	CGCGCCCTTGTCGTCATCGTCTTTGTAGTCGATGTCATGATCTTTATAATCACCGTCATGGTCTTTGTAGTC
pUAS.K10-MS2.NLS.3XFLAG.HAT	CBPhatK10Fw	TATT***GGCGCGCC***ACAATTCAATCCCGAGGAACTGCG
	CBPhatRw	ATCT***TCTAGA***TCAGTCCTGACCCTGGTTATGCAG
pUAS.K10-MS2.NLS.3XFLAG.HAT F2161A	CBPhatK10Fw	TATT***GGCGCGCC***ACAATTCAATCCCGAGGAACTGCG
	CBPhatRw	ATCT***TCTAGA***TCAGTCCTGACCCTGGTTATGCAG
pUAS.K10-dCas9.NLS.HAT	CBPhatK10Fw	TATT***GGCGCGCC***ACAATTCAATCCCGAGGAACTGCG
	CBPhatRw	ATCT***TCTAGA***TCAGTCCTGACCCTGGTTATGCAG
pUAS.K10-dCas9.NLS.HAT F2161A	CBPhatK10Fw	TATT***GGCGCGCC***ACAATTCAATCCCGAGGAACTGCG
	CBPhatRw	ATCT***TCTAGA***TCAGTCCTGACCCTGGTTATGCAG

Table S3. Comparison of histone acetylation in S2 cells and early embryos

Gene	H3K27ac in S2 cells				H3K27ac in 0-4h embryos			
	**promoter**	**enhancer**	**enhancer**	**enhancer**	**promoter**	**enhancer**	**enhancer**	**enhancer**
twi	-	+			-	+		
wg	-	++	++	-	-	-	+	+
en	-	-	-		-	-	-	
AttC	-				-			
sna	-				-			
hnt	+				-			
sog	+	++			+	-		
CG42741	-				+			
Fatp2	-				-			
inv	-				-			
Wnt4	-				-			
Wnt6	+				-			
CG43691	+				-			
CG4744	-				-			
